# Supplementary material for: Green synthesis and characterization of silver nanoparticles and its efficacy against Rhizoctonia solani, a fungus causing sheath blight disease in rice
Source: PLoS One. 2024 Jun 18;19(6):e0304817. doi: 10.1371/journal.pone.0304817 (PMC11185457; doi:10.1371/journal.pone.0304817)

PLOS ONE

Green synthesis and characterization of silver nanoparticles and its efficacy against Rhizoctonia solani, a fungus causing sheath blight disease in rice

--Manuscript Draft--

**Manuscript Number:**	PONE-D-23-39382
**Article Type:**	Research Article
**Full Title:**	Green synthesis and characterization of silver nanoparticles and its efficacy against Rhizoctonia solani, a fungus causing sheath blight disease in rice
**Short Title:**	Green synthesis of silver nanoparticles and its efficacy against sheath blight disease of rice.
**Corresponding Author:**	M. A. Latif<br>Bangladesh Rice Research Institute<br>Gazipur, Joydebpur BANGLADESH
**Keywords:**	Characterization, Nanoparticles, Rice disease, Sheath blight, Silver Nano.
**Abstract:**	Sheath blight is one of the devastating menaces of rice production in the world. Nano technology opens an era to create nano scale composite for effective management of plant diseases. In this study, silver nano particles (AgNPs) were synthesized using AgNO3 (Ag) and Dholkolmi (D) (I. carnea) leaf extract. pH was adjusted to 9, 10 and 11 to signify the production of AgNPs. AgNPs was were characterized using UV-vis spectrophotometer, X-ray Diffraction (XRD), Fourier Transform Infrared Spectroscopy (FTIR), Particle Size Analyzer, Zeta potential, Scanning Electron Microscope (SEM), Field Emission Scanning Electron Microscope (FESEM), and Transmission Electron Microscope (TEM). Additionally, the antifungal activity of AgNPs against R. solani was tested in vitro and in vivo conditions. The results of UV-visible spectrophotometers showed an absorption peak ranging from 421 to 434 nm, validating the synthesis of AgNPs in the solution. XRD, DLS and TEM estimated AgNPs sizes are ~45 nm, 66.2nm and 46.38 to 73.81 nm, respectively. SEM and FESEM demonstrated that the synthesized AgNPs were spherical in shape. Strong silver signals and weak oxygen, aluminium, and carbon peaks in the EDX profile were indicative of the presence of other biomolecules attached to the surface of the AgNPs. Among the different treatments AgNPs produced from the ration Ag:D=10:1 (pH10) showed the most significant mycelium growth reduction (1.2 cm) over the control (8.7 cm). Over time (0 hour to 72 hour) mycelium growth inhibition was increased significantly in most of the AgNPs treatments. In every time interval Amister Top (91 to 97% mycelium inhibition), AgNO3 (62 to 86% inhibition) and AgNO3:Dholkolimi leaf extract=10:1(pH10) (70 to 89% inhibition) performed well compared to other treatments. In the pot experiments, significant disease inhibition (15.40% and 13.27% RLH) was noticed in AgNPs at 7.5ppm and 37.5ppm compared to disease control (23.12% and 24.98% RLH, respectively). In the field, the highest percentage of RLH was recorded in disease control (64.25%) and the lowest RLH was found in Amister Top (23.65% RLH) followed by 11.25ppm AgNPs (34.03% RLH), 7.5ppm AgNPs (36.80% RLH), 6ppm AgNPs (44.39% RLH) and 11.25ppm AgNO3 (51.92% RLH). Results of the in vitro and in vivo experiments provided a piece of evidence that AgNPs have a potent antifungal effect and it could be used as a potential agent to control sheath blight disease of rice.
**Order of Authors:**	A.K.M. Sahfiqul Islam
	Md. Rejwan Bhuiyan
	Sheikh Arafat Islam Nihad
	Mohammad Ashik Iqbal Khan
	Rumana Akter
	Shamima Akter
	Md. Rashidul Islam
	Md. Atiqur Rahman Khokon

Powered by Editorial Manager® and ProduXion Manager® from Aries Systems Corporation

	Mohammad Abdul Latif
**Additional Information:**	
**Question**	**Response**
**Financial Disclosure**<br><br>Enter a financial disclosure statement that describes the sources of funding for the work included in this submission. Review the submission guidelines for detailed requirements. View published research articles from *PLOS ONE* for specific examples.<br><br>This statement is required for submission and **will appear in the published article** if the submission is accepted. Please make sure it is accurate.<br><br>**Funded studies**<br>Enter a statement with the following details:<br>• Initials of the authors who received each award<br>• Grant numbers awarded to each author<br>• The full name of each funder<br>• URL of each funder website<br>• Did the sponsors or funders play any role in the study design, data collection and analysis, decision to publish, or preparation of the manuscript?<br><br>Did you receive funding for this work?	Yes
Please add funding details.<br>as follow-up to "**Financial Disclosure**<br><br>Enter a financial disclosure statement that describes the sources of funding for the work included in this submission. Review the submission guidelines for detailed requirements. View published research articles from *PLOS ONE* for specific examples.<br><br>This statement is required for submission and **will appear in the published article** if the submission is accepted. Please make sure it is accurate.	This experiment was conducted under the project titled “Sustainable management of blast, sheath blight and bacterial blight diseases of rice through Nanoparticles (Project ID:TF 71-C/20) funded by Krishi Gobeshona Foundation (KGF), Ministry of Agriculture, Bangladesh.

Powered by Editorial Manager® and ProduXion Manager® from Aries Systems Corporation

**Funded studies**<br>Enter a statement with the following details:<br>• Initials of the authors who received each award<br>• Grant numbers awarded to each author<br>• The full name of each funder<br>• URL of each funder website<br>• Did the sponsors or funders play any role in the study design, data collection and analysis, decision to publish, or preparation of the manuscript?<br><br>Did you receive funding for this work?"	
Please select the country of your main research funder (please select carefully as in some cases this is used in fee calculation).<br>as follow-up to "**Financial Disclosure**<br><br>Enter a financial disclosure statement that describes the sources of funding for the work included in this submission. Review the submission guidelines for detailed requirements. View published research articles from *PLOS ONE* for specific examples.<br><br>This statement is required for submission and **will appear in the published article** if the submission is accepted. Please make sure it is accurate.<br><br>**Funded studies**<br>Enter a statement with the following details:<br>• Initials of the authors who received each award<br>• Grant numbers awarded to each author<br>• The full name of each funder<br>• URL of each funder website<br>• Did the sponsors or funders play any role in the study design, data collection and analysis, decision to publish, or preparation of the manuscript?<br><br>Did you receive funding for this work?"	BANGLADESH - BD
**Competing Interests**	The authors have declared that no competing interests exist.

Use the instructions below to enter a competing interest statement for this submission. On behalf of all authors, disclose any competing interests that could be perceived to bias this work—acknowledging all financial support and any other relevant financial or non-financial competing interests.<br><br>This statement is required for submission and **will appear in the published article** if the submission is accepted. Please make sure it is accurate and that any funding sources listed in your Funding Information later in the submission form are also declared in your Financial Disclosure statement.<br><br>View published research articles from *PLOS ONE* for specific examples.<br><br>**NO authors have competing interests**<br><br>Enter: *The authors have declared that no competing interests exist.*<br><br>**Authors with competing interests**<br><br>Enter competing interest details beginning with this statement:<br><br>*I have read the journal's policy and the authors of this manuscript have the following competing interests: [insert competing interests here]*<br><br>* typeset	
**Ethics Statement**<br><br>Enter an ethics statement for this submission. This statement is required if the study involved:<br><br>• Human participants<br>• Human specimens or tissue<br>• Vertebrate animals or cephalopods<br>• Vertebrate embryos or tissues	N/A

• Field research<br><br>Write "N/A" if the submission does not require an ethics statement.<br><br>General guidance is provided below. Consult the submission guidelines for detailed instructions. **Make sure that all information entered here is included in the Methods section of the manuscript.**	

**Format for specific study types**<br><br>**Human Subject Research (involving human participants and/or tissue)**<br>• Give the name of the institutional review board or ethics committee that approved the study<br>• Include the approval number and/or a statement indicating approval of this research<br>• Indicate the form of consent obtained (written/oral) or the reason that consent was not obtained (e.g. the data were analyzed anonymously)<br><br>**Animal Research (involving vertebrate animals, embryos or tissues)**<br>• Provide the name of the Institutional Animal Care and Use Committee (IACUC) or other relevant ethics board that reviewed the study protocol, and indicate whether they approved this research or granted a formal waiver of ethical approval<br>• Include an approval number if one was obtained<br>• If the study involved *non-human primates*, add *additional details* about animal welfare and steps taken to ameliorate suffering<br>• If anesthesia, euthanasia, or any kind of animal sacrifice is part of the study, include briefly which substances and/or methods were applied<br><br>**Field Research**<br><br>Include the following details if this study involves the collection of plant, animal, or other materials from a natural setting:<br>• Field permit number<br>• Name of the institution or relevant body that granted permission	
**Data Availability**<br><br>Authors are required to make all data underlying the findings described fully available, without restriction, and from the time of publication. PLOS allows rare exceptions to address legal and ethical concerns. See the PLOS Data Policy and FAQ for detailed information.	Yes - all data are fully available without restriction

A Data Availability Statement describing where the data can be found is required at submission. Your answers to this question constitute the Data Availability Statement and **will be published in the article**, if accepted.<br><br>**Important:** Stating 'data available on request from the author' is not sufficient. If your data are only available upon request, select 'No' for the first question and explain your exceptional situation in the text box.<br><br>Do the authors confirm that all data underlying the findings described in their manuscript are fully available without restriction?	
**Describe where the data may be found in full sentences. If you are copying our sample text, replace any instances of XXX with the appropriate details.**<br><br>• If the data are **held or will be held in a public repository**, include URLs, accession numbers or DOIs. If this information will only be available after acceptance, indicate this by ticking the box below. For example: *All XXX files are available from the XXX database (accession number(s) XXX, XXX.*).<br>• If the data are all contained **within the manuscript and/or Supporting Information files**, enter the following: *All relevant data are within the manuscript and its Supporting Information files.*<br>• If neither of these applies but you are able to provide **details of access elsewhere**, with or without limitations, please do so. For example:<br><br>*Data cannot be shared publicly because of [XXX]. Data are available from the XXX Institutional Data Access / Ethics Committee (contact via XXX) for researchers who meet the criteria for access to confidential data.*<br><br>*The data underlying the results presented in the study are available from (include the name of the third party*	All relevant data are within the manuscript and its Supporting Information files.

Powered by Editorial Manager® and ProduXion Manager® from Aries Systems Corporation

and contact information or URL).<br>• This text is appropriate if the data are owned by a third party and authors do not have permission to share the data.<br><br>* typeset	
Additional data availability information:	

Powered by Editorial Manager® and ProduXion Manager® from Aries Systems Corporation

Manuscript

Click here to access/download;Manuscript;Islam et al.,2023 manuscript.docx

Green synthesis and characterization of silver nanoparticles and its efficacy against *Rhizoctonia solani*, a fungus causing sheath blight disease in rice

A.K.M. Sahfiqul Islam[1] (sahfiqul34@gmail.com), Rejwan Bhuiyan[1] (rejwanbrri@gmail.com), Sheikh Arafat Islam Nihad[1] (nihad402@gmail.com), Rumana Akter[1] (rumana2252@gmail.com), Mohammad Ashik Iqbal Khan[1] (ashikjp@gmail.com), Shamima Akter[1] (sakter140@gmail.com), Md. Rashidul Islam[2] (rashidul.islam@bau.edu.bd), Md. Atiqur Rahman Khokon[1] (atiq.ppath@bau.edu.bd), Mohammad Abdul Latif [1] (alatif1965@yahoo.com)

[1]Plant Pathology Division, Bangladesh Rice Research Institute (BRRI), Gazipur-1701, Bangladesh.

[2]Department of Plant Pathology Division, Bangladesh Agricultural University, Mymensingh-2202, Bangladesh.

Short title: Green synthesis of silver nanoparticles and its efficacy against sheath blight disease of rice.

Corresponding Author

Dr. Mohammad Abdul Latif
Director (Admin and common service),
Former Head and Chief Scientific Officer,
Plant Pathology Division,
Bangladesh Rice Research Institute (BRRI)
Phone: +8801715034094
E-mail: latifbrri@gmail.com, alatif1965@yahoo.com
ORCID id: https://orcid.org/0000-0002-4289-575X

Abstract

Sheath blight is one of the devastating menaces of rice production in the world. Nano technology opens an era to create nano scale composite for effective management of plant diseases. In this study, silver nano particles (AgNPs) were synthesized using AgNO3 (Ag) and Dholkolmi (D) (*I. carnea*) leaf extract. pH was adjusted to 9, 10 and 11 to signify the production of AgNPs. AgNPs was were characterized using UV-vis spectrophotometer, X-ray Diffraction (XRD), Fourier Transform Infrared Spectroscopy (FTIR), Particle Size Analyzer, Zeta potential, Scanning Electron Microscope (SEM), Field Emission Scanning Electron Microscope (FESEM), and Transmission Electron Microscope (TEM). Additionally, the antifungal activity of AgNPs against *R. solani* was tested in vitro and in vivo conditions. The results of UV-visible spectrophotometers showed an absorption peak ranging from 421 to 434 nm, validating the synthesis of AgNPs in the solution. XRD, DLS and TEM estimated AgNPs sizes are ~45 nm, 66.2nm and 46.38 to 73.81 nm, respectively. SEM and FESEM demonstrated that the synthesized AgNPs were spherical in shape. Strong silver signals and weak oxygen, aluminium, and carbon peaks in the EDX profile were indicative of the presence of other biomolecules attached to the surface of the AgNPs. Among the different treatments AgNPs produced from the ration Ag:D=10:1 (pH10) showed the most significant mycelium growth reduction (1.2 cm) over the control (8.7 cm). Over time (0 hour to 72 hour) mycelium growth inhibition was increased significantly in most of the AgNPs treatments. In every time interval Amister Top (91 to 97% mycelium inhibition), $AgNO_3$ (62 to 86% inhibition) and $AgNO_3$:Dholkolimi leaf extract=10:1(pH10) (70 to 89% inhibition) performed well compared to other treatments. In the pot experiments, significant disease inhibition (15.40% and 13.27% RLH) was noticed in AgNPs at 7.5ppm and 37.5ppm compared to disease control (23.12% and 24.98% RLH, respectively). In the field, the highest percentage of RLH was recorded in disease control (64.25%) and the lowest RLH was found in Amister Top (23.65% RLH) followed by 11.25ppm AgNPs (34.03% RLH), 7.5ppm AgNPs (36.80% RLH), 6ppm AgNPs (44.39% RLH) and 11.25ppm $AgNO_3$ (51.92% RLH). Results of the in vitro and in vivo experiments provided a piece of evidence that AgNPs have a potent antifungal effect and it could be used as a potential agent to control sheath blight disease of rice.

Keywords: Characterization, Nanoparticles, Rice disease, Sheath blight, Silver Nano.

1. Introduction

Rice (*Oryza sativa*) is an ancient and one of the most ideal staple foods for human beings. Wide range of adaptability to different ecosystems from hilly area to submergence make it more acceptable over other crops. Even if diets are becoming more varied these days, rice still accounts for over half of the world's daily caloric intake and is therefore the most important staple meal for over half of the human population [1,2]. Research indicates that the worldwide population is expected to rise by 7 to 10 billion people by 2050, leading to a significant rise in the demand for rice, even with the assurance that rice yields will increase by 25% by 2030 [2]. Bangladesh is a large contributor to global rice production and it ranked as the third rice producing country in the world. Over the last 30 years, rice production in Bangladesh has significantly increased across the country [3]. Intensive rice cultivation and high input requirements make the high-yielding varieties vulnerable to different biotic stresses [4]. So, far 32 rice diseases have been identified in Bangladesh and among them blast, sheath blight, bacterial blight, false smut and tungro are the major diseases of rice in the country [5–9].

Sheath blight caused by *Rhizoctonia solani* signifies its importance due to its severity and lack of resistant source in the country as well as in the world [9]. Sheath blight of rice is a very destructive disease that results in quality degradation and up to a 50% yield loss each year throughout the world [10–12]. Only a small number of fungicides are available for the management of rice sheath blight, and chemical control is the primary method of disease management [13]. Numerous fungicides, including benomyl, captafol, carbendazim, carboxin, chloroneb, edifenphos, mancozeb, thiophanate, and zineb, were successful in controlling the disease in the field [14,15]. However, the excessive and mischievous use of these fungicides could result in developing resistance in the pathogen [16] and causes long-term harm to the environment and human health [17]. However, to ensure environmental safety there is a need for new fungicides with low toxicity, high selectivity and high activity against fungal strains that are resistant to other fungicides. The approach for discovery of new fungicides and new formulations against rice sheath blight is of vital importance.

Nanotechnology represents a possible new management tool, since nanoparticles have been reported to have microbial inhibitory effects [18]. Nanoparticles are characterized by their particle size, which generally ranges between 1 to 100 nm [19,20]. In recent years, the use of nanomaterials has had a significant impact on several high-tech industries, including medication

delivery, cancer therapy [21], energy and biomedicine [22] agriculture [23], and many other sectors [24]. Using atomic-scale materials, new methods of disease control have been made possible by nanotechnology [25]. Due to their enormous surface to volume ratio, which offers a large contact surface with pathogen sources, very small-scale particles have emerged as contemporary agents [26]. Small-scale tools [27], plant protection products [28] fertilizers [29], water purification and pollutant remediation [30], nano sensors, diagnostic tools [31], and plant genetic modification [32] are just a few examples of how nanotechnology can have a significant impact on natural processes and agriculture.

It is well known that silver nanoparticles (AgNPs) are widely used in fields such as photonics, catalysis, bio-nanomaterials, and medicine due to its inherent morphology, composition, and crystal structure [33–35]. AgNPs are highly reactive and have potential inhibitory effects against bacteria and fungi [36,37]. They can absorb quickly into cells, even at low concentrations resulting death of that microorganism [37]. In large-scale biological processes, AgNPs can damage microorganisms, including the cell membrane structure [38,39]. The basis for the antibacterial effect of silver ionsis to pass through the bacterial cell wall and to alter cellular signaling [40]. AgNPs with fungistatic, bacteriostatic, and plasmonic characteristics are among the more environmentally friendly plant pathogen inhibitors when compared to synthetic fungicides [41]. AgNPs inhibit the mycelium growth of phytopathogenic fungi including *Alternaria alternata*, *Botrytis cinerea*, and *Colletotrichum gloeosporioides* [42]. Therefore, the antifungal activity of AgNPs has gotten less attention than it has in the medical and pharmaceutical sciences.

At present, the preparation methods used for AgNPs are mainly physical, chemical, and/or biological methods [43]. The wide dispersion, high purity, and strong activity of the nano-silver particles produced by physical methods come at the cost of using high pressure and temperature conditions during the procedure. Because of the high energy consumption and tendency of the produced particles to aggregate, AgNPs operate less effectively [44]. Although the chemical method can yield the AgNPs with a narrow particle size distribution range and difficult to agglomerate, the reaction process employs strong reducing agents such as hydrazine, dimethylformamide, sodium borohydride, and other toxic organic reagents, which pose a serious threat to the environment [45,46].

In recent years, due to the increasing concern towards the subject of green chemistry, by virtue of it being simple, efficient, and pollution-free, the biological method with microorganisms and plants has attracted the interest of researchers [47]. Due to their ecologically benign character,

plant systems have been utilized as a dependable, green technique for the production of nanoparticles [43]. This method is easier to use, employs mild conditions, takes less time to complete, and has easy access to ingredients, making it more ideal for the production of AgNPs [45]. It has been reported that the plant materials can be used to synthesize AgNPs, such as *Ficus carica*, *Coffea arabica*, *Rosmarinus officinlis*, *Lawsonia inermis*, *Aloe vera*, *Eclipta alba*, *Momordica charantia*, *Leptadenia reticulataand* and so on [48,49].

Considering the advantage of green synthesizing of nanoparticles, this study was undertaken to develop silver nano particles (AgNPs) using $AgNO_3$ and *I. carnea* (Dholkolmi) leaf extract. To confirm and characterize the AgNPs, different methods were applied in this experiment. Finally, we conducted in vitro and in vivo experiment to test the efficacy AgNPs against *R. solani* causing sheath blight disease in rice.

2.Materials and methods

2.1. Preparation of Leaf Extracts

Dholkolmi (*Ipomoea carnea*) leaf was used as reducing agents to biosynthesize silver nano-particles (AgNPs). This plant was selected due to its low cost, convenience of availability, and therapeutic qualities. From the territory of the Bangladesh Rice Research Institute in Gazipur, fresh dholkolmi leaves were collected. These were properly cleaned under running water to get rid of dirt and other organic impurities, washed with distilled water a couple of times, and then let to air dry at room temperature. A beaker containing 20 g of finely chopped leaves and 300 mL of distilled water was heated to 80°C and then boiled for 30 minutes. The extract was cooled down and twice filtered using Whatman No. 1 filter paper to get rid of contaminants and get clean solutions.

2.2. Biosynthesis of AgNPs

Silver nitrate ($AgNO_3$) solution with 2mM concentration was prepared for the manufacture of AgNPs. Three distinct leaf extract concentrations (1 mL, 2 mL, and 3 mL) were employed in these trials. Each leaf extract was applied under four distinct conditions, including fresh leaves (pH-5.88), leaf extracts at pH 9, 10 and 11. The pH of the leaf extract was adjusted to 9, 10 and 11 and mixed with 10mL solution of 2 mM $AgNO_3$ at three different ratios viz. 1:10, 2:10 and 3:10 (Dholkolmi leaves extract (D): $AgNO_3$). After mixing, a distinct color transformation (from light orange to dark brown) was seen, indicating the biosynthesis of AgNPs (Figure 1).

2.3. Characterization of AgNPs

The samples were characterized by different analytical methods using sophisticated instruments for observing the optical properties of biosynthesized nanoparticles. The samples were initially analyzed using UV–vis spectrophotometer (Model Shimadzu UV- 2600i, Japan) to detect the presence of AgNPs at a specific wavelength. To observe the crystalline and amorphous nature of AgNPs, X-ray diffraction (XRD) evaluations of AgNPs' powder were carried out by Smart Lab, Rigaku, Japan. By employing Cu K radiation (0.154nm) at a scanning rate of 30.0°/min ranging from 5° to 90° and an X-ray tube operating at 40kV and 50mA, XRD patterns were captured. To examine whether functional groups were present on the surface of nanoparticles, Fourier Transform Infrared Spectroscopy (FTIR; Perkin Elmer, Spectrum II) was utilized. The oven dried AgNPs powder was subjected to FTIR spectroscopy measurements. Particle size distribution of AgNPs were observed by Dynamic Light Scattering (DLS) method and the zeta potentials were determined using Litesizer 500 (Anton Paar, United Kingdom). Scanning Electron Microscope (SEM) observation was done byJCM7000, Jeol, Japan at the Plant Pathology Division lab of Bangladesh Rice Research Institute (BRRI), Gazipur-1701. The experiment was carried out at a 15 KV accelerating voltage. After applying a gold coating on the slide, a SEM picture was obtained. Using an EDX spectrometer, the elemental makeup of the produced nanoparticles was identified. Additionally, to capture the quality structure of AgNPs, the JEOL-7600F was used to do Field Emission Electron Microscope (FESEM) study.

2.4. Antifungal activity of AgNPs

Different concentrations of AgNPs with the following ration were prepared to test the efficacy of silver nano solutions. A total of 17 treatments i.e., $AgNO_3$:D (Dholkolmi)=10:1, $AgNO_3$:D=10:2, $AgNO_3$:D=10:3, $AgNO_3$:D=10:1(pH9), $AgNO_3$:D=10:2(pH9), $AgNO_3$:D=10:3(pH9), $AgNO_3$:D=10:1(pH10), $AgNO_3$:D=10:2(pH10),$AgNO_3$:D=10:3(pH10), $AgNO_3$:D=10:1(pH11), $AgNO_3$:D=10:2(pH11), $AgNO_3$:D=10:3(pH11), $AgNO_3$ (0.002%), Amister Top (1 ppm), Disease control, Leaf extract and Water were mixed individually with Potato Dextrose Agar (PDA) media (poisoned food method) to assess the in vitro antifungal effects of AgNPs against *R. solani*. In the middle of each Petri plate, 5 mm-diameter agar plugs with fungus mycelia were positioned and incubation for three days at 28°C. The growth inhibition rate of mycelia was calculated using the following equation Inhibition rate (RH, %) = ((R-r)/R)*100 (Khatami et al 2015), Where R is the fungal mycelia's radial growth on the control plate and r is the mycelia's radial growth on the AgNPs-treated plate. Additionally, mycelium

growth was measured at four time interval (0 hr, 24 hr, 48hr and 72 hr) to observe the rate of fungal growth in respective of each treatment.

2.5. Determination of AgNPs concentration

To apply AgNPs in vivo experiment, AgNPs concentration was measured from the solutions of $AgNO_3$ and Dholkolmi leaf extracts. Spectrophotometer is used to determine the concentration of the prepared silver nanoparticles and the results were derived from the calibration curve based on the linear standard curve equation ($y = 0.0512x + 0.0079$; $R^2 = 0.997$) of standard silver nanoparticles.

2.6. *In vivo* examination of AgNPs on sheath blight disease under greenhouse condition

Two pot experiment were conducted to evaluate the efficacy of AgNPs against sheath blight disease. Thirty days old seedlings of highly susceptible variety BRRI dhan48 were sown in each pot to conduct the experiment. Randomized Complete Block Design (RCBD) with three replication of each treatment was followed to conduct the study. For the first pot experiment, a total of 10 treatments i.e. $AgNO_3$ (7.5ppm), 1.5ppm, 3ppm, 4.5ppm, 6ppm, 7.5ppm AgNPs solution, Amister Top, Disease control, Dholkolmi leaf extracts and Healthy control were used in in-vivo experiment. The 3 day old mycelial blocks *R. solani* were placed in the center of the plants at maximum tillering stage right above the water line and observed for sheath blight disease development. The first and second sprays were applied to each treatment after two and ten days after inoculation, respectively. Sheath blight disease severity was assessed 21 days after disease inoculation and relative lesion heigh was calculated using the following formula: Relative lesion heigh (RLH) = (Lesion height/Plant height)*100. Based on the first pot experiment result, second pot experiment was setup similarly by increasing the concentration of the treatments with expectation of better performance of AgNPs. The treatments of second experiment was $AgNO_3$ (37.5ppm), 7.5ppm, 15ppm, 22.5ppm, 30ppm, 37.5ppm of AgNPs solution, Disease control, Dholkolmi leaf extracts and Healthy control.

2.7. Effect of AgNPs under field conditions on sheath blight disease of rice

The experiment was carried out in the experimental field of Plant Pathology Division of BRRI. A sheath blight susceptible rice variety BR11 was used for field experiment. The plot size

was 3m × 3m and a randomized complete block design with three replications was used to carry out the study. Thirty days old seedlings were transplanted at a 20 cm to 20 cm spacing and recommended agronomic practices were maintained during the study. Rice plants were inoculated with 3 days old culture of *R. solani* at maximum tillering stage. Eight treatments (T1: Healthy control, T2: Disease control, T3: Leaf extract (Dholkolmi), T4: $AgNO_3$ (11.25ppm), T5: Amister Top (1ppm), T6: 6ppm AgNPs, T7: 7.5ppm AgNPs and T8: 11.25ppm of AgNPs solution were sprayed two times in each plot. First spray and second spray were applied after 3 days and 10 days after inoculation, respectively.

3. Results

3.1. Biosynthesis and characterization of AgNPs

3.1.1. Visual observation

The silver nitrate solution began to change color when the dholkolmi leaf extract was added. After 30 minutes of constant stirring, the solution gradually darkened. The color of reaction mixtures changed from being light orange to reddish brown indicated the production of AgNPs in the solution (Figure 1). Leaf extracts concentration and pH of the leaf extracts played a vital role for the synthesizing AgNPs. The colour of the reaction becomes darkened when pH and concentration of the leaf extract increased (Figure 1), suggesting that the more production of AgNPs in the solution. After 30 min, the solution of the 3 mL leaf extract at pH11 caused the rapid change in color from light orange to dark brown, indicating the fast reduction of Ag^+ to Ag^0 in AgNO3 solution, while the color in other samples was changed after incubation for 0.3–8 h in a dark condition and the control sample remained light orange colour.

Figure 1. Color change after AgNPs formation (a-d) and UV-Vis spectra showing absorbance with different concentration and pH of plant extract (e-h).

3.1.2. UV–vis absorbance studies

The results of UV-visible spectrophotometer showed a spectrum of surface plasmon resonance (SRP) with an absorption band ranging from 421 to 434 nm, validated the synthesis of AgNPs in the solution (Figure 1). Indeed, a stronger and sharper absorption band at 433 nm was visible in the SPR spectra of AgNPs produced from the increased concentration of leaf extract (Figure 1h). Additionally, the UV-vis spectra results revealed that the reaction mixture's absorbance intensity increased with time, leaf extract concentration, and pH.

3.1.3.X-ray diffraction (XRD)

X-ray diffraction (XRD) analysis was used to analyze the AgNPs from dholkolmi leaf extracts (Figure 2a). The XRD data were compared to the database of the Joint Committee on Powder Diffraction Standards (JCPDS, file nos. 04-0783) and the pure crystalline silver structure. Four intense diffraction peaks at 2θ values of 38.04°, 46.13°, 64.43°, and 76.61° were indexed to the (003), (002), (019) and (008) planes of silver reflections, respectively. The average crystallite sizes of the nanoparticles were calculated by using Debye-Scherrer's equation: $D=K\lambda/\beta \cos \theta$, where, D is the estimated crystal size from XRD patterns, θ is the Bragg angle in degrees, λ is the wavelength of X-ray source used, β is the angular width at the half maximum of the diffraction peak and K is the constant of Debye-Scherrer's equation. The estimated crystalline size of the biosynthesized silver nanoparticles is found to be ~45 nm. Moreover, the XRD patterns confirmed that the silver nanoparticles are of face centered cubic crystalline nature.

Figure 2. X-Ray Diffraction pattern (a), FTIR spectrum (b), particle size diameter (c) and Zeta potential (d) of AgNPs

3.1.4. FTIR of silver nano-particles

Analyzing the silver nanoparticle using FTIR made it possible to validate the existence of specific functional groups and the plant extract's dual role as a capping and reducing agent. The wave number of free hydroxyl groups and phenol groups significantly changed (3681cm^{-1}), indicating that polyphenol-containing alkaloids may be involved in the reduction of silver nitrate and further stabilization of AgNPs (Figure 2b). It's interesting to note that the wave number (666 cm^{-1}) that corresponds to sulphonic groups also changed significantly. 3.1.5. Particle size distribution of AgNPs.

3.1.5. DLS and Zeta potential

According to the DLS pattern, this approach produces silver nanoparticles with a diameter that ranges from 12.3 nm to 110.5 nm (Figure 2c) with polydispersity index (PDI) of 0.25. The average diameter of the particles was measured to be 66.2nm. Zeta potential is provided as a measure of the stability of the solution; the larger the absolute value, the stronger the electrostatic repulsion. The biosynthesized AgNPs' zeta potential in water was measured to check the stability of synthesized AgNPs. The zeta potential of AgNPs by dholkolmi leaf extract in the current investigation was -27.1 mV at pH 10.0 (10:1 ratio) (Figure 2d). The formulation becomes more stable as a result of the large negative value, which reinforces the repulsion between the particles.

3.1.6. SEM and FESEM with EDX of AgNPs

The high-density silver nanoparticles synthesized by the *I. carnea* leaf extract was visible in the SEM and FESEM (Figures 3a-b), which demonstrated the polydispersed AgNPs of various sizes. The particles were spherical in shape. Strong silver signal and weak oxygen, aluminum, and carbon peaks in the EDX profile were indicative of the presence of biomolecules attached to the surface of the AgNPs (Figure 3c).

Figure 3. SEM (a), FESEM (b), EDX spectra (c), and TEM (d) images of biosynthesized AgNPs.

3.1.7. TEM Analysis

The size, form, and morphology of nanoparticles have all been determined using transmission electron microscopy (TEM). The produced silver nanoparticles in TEM micrograph were found to be correctly distributed, spherical, and ranged in size from 46.38 to 73.81 nm with a few particles being bigger than average due to nanoparticle coagulation (Figure 3d).

3.2. *In vitro* examination of inhibitory effects of AgNPs on mycelial growth of *R. solani*

The antifungal potential of biosynthesized silver nanoparticles was evaluated in the current study utilizing the poisoned food approach against *R. solani* and compared with the control (water, $AgNO_3$ (0.002%), Amister Top (1ppm), Leaf extract). The findings showed that the in vitro growth of *R. solani* was considerably suppressed by both the amister top (0.4 cm) and $AgNO_3$:Dholkolimi leaf extract=10:1 (pH 10) (1.2 cm) treatments. The effect of tested AgNPs with different concentrations on mycelial growth are presented in Figure 4. Among the different treatments of Dholkolmi leaf extract (D) mediated AgNPs, Ag:D=10:1 (pH 10) showed the most significant mycelium growth reduction (1.2 cm) over control (8.7cm). Over the time (0 hour to 72 hour) mycelium growth inhibition was significantly increased in most of the treatments (Figure 5). In every time interval Amister Top (91 to 97% mycelium inhibition), $AgNO_3$ (62 to 86% mycelium inhibition) and $AgNO_3$:Dholkolimi leaf extract=10:1(pH10) (70 to 89% mycelium inhibition) performed well compared to other treatments (Figure 5).

Figure 4. Efficacy of synthesized AgNPs against *R. solani* mycelium growth inhibition in in vitro condition. Here, (a) Fungicide, (b) AgNO3, (c) AgNPs, (d) Disease control (*R. solani*), (e) Leaf extract and (f) Water (g) effect of dholkolmi leaf extract mediated AgNPs on mycelial growth of *R. solani*. Here, Ag= AgNO3, D = Dholkolmi leaves extract.

Figure 5. Hourly efficacy of AgNPs at different concentration and pH to control the mycelium growth of *R. solani* at invitro condition.

3.3. *In vivo* examination of AgNPs on sheath blight disease under greenhouse condition

Different concentrations Dholkolmi leaf extract mediated AgNPs were tested against *R. solani* for the management of sheath blight disease (Figure 6). Varying levels of inhibitory effects were found in plants treated with AgNPs. In first pots experiment, highest disease inhibition (15.40% RLH) was noticed in AgNPs (7.5ppm) which was significant compared to disease control (23.12%) (Figure 6a). In second pot experiments, similar pattern was found and significant reduction (13.27% RLH) was found in AgNPs (37.5ppm) compared to disease control (24.98% RLH) (Figure 6b). In both Pot experiments, higher dose of AgNPs had no significant difference with Amister Top (Figures 6a and b) which providing evidence that AgNPs has a potent antifungal effect and reduces lesion in the rice tillers.

Figure 6. Efficacy of AgNPs against sheath blight disease of rice in pot (a-b) and field experiments (c).

3.4. Effect of AgNPs on sheath blight disease under field condition

The effect of different treatments on sheath blight at field condition are shown in Figure 6c. The highest percent of RLH was recorded in disease control (64.25%) and the lowest RLH was found in Amister Top (23.65% RLH) followed by 11.25ppm AgNPs (34.03% RLH), 7.5ppm AgNPs (36.80% RLH), 6ppm AgNPs (44.39% RLH) and 11.25ppm $AgNO_3$ (51.92% RLH) (Figure 6c). A field reaction of AgNPs against sheath blight disease are shown in Figure 7.

Figure 7. Sheath blight disease suppression due to application of AgNPs application. Here, (a) disease control and (b) AgNPs treated.

4. Discussion

Nano technology is one of the most promising advanced ways for controlling crop diseases. Albeit its vast potential, application of nanoparticles to control rice diseases are very few. Green synthesizing of AgNPs and its characterization and in-vitro, in-vivo and field application of AgNPs for controlling sheath blight disease make our study unique compared to other experiments. Posi-

tive finding of AgNPs for controlling sheath blight disease signify the potentiality of the nanoparticles application to control rice diseases. Nevertheless, the results of this study offer important information about the effectiveness of AgNPs for the treatment of the rice sheath blight disease caused by *R. solani*. The results indicate that *R. solani* can be inhibited by AgNPs, but the outcomes depend on the concentration of AgNPs used to treat the pathogen.

The present study revealed that *I. carnea* leaf extracts acted as a reducing agent in green synthesis of AgNPs. Color changes throughout the synthesis process can be used to identify the creation of AgNPs. AgNPs had a reddish-brown color in aqueous solution because surface plasmon vibrations were excited [50]. The simplest method for basic characterization of AgNPs was this visual observation. According to earlier research on the phytochemistry of *I. carnea*, the main chemical components included calystegineB2, calystegineC1, and swainsnine alkaloids. As a result, polyphenols might have a role in the conversion of silver nitrate to AgNPs [51].

In our study, increased of pH and volume of leaf extracts transformed the reaction color from light orange color to dark brown and it indicated the production of AgNPs in the solutions. pH and the concentration of the reactants both affect how intense the resulting color is [44]. The shape and size of the particles were affected by pH because it had the ability to alter the charge of biomolecules, which might have an effect on both their capping and stabilizing capabilities [52]. The pH of the solution had a substantial impact on the size, shape, and optical properties of the nanoparticles [53]. In addition, it was discovered that the pH accelerated the reduction reaction as seen by how soon the solution turned into dark brown when $AgNO_3$ and aqueous Dholkolmi leaf extract were combined at pH 11. The stimulation of silver nanoparticles' surface plasmon oscillations causes AgNPs to appear brown in aqueous solution [54]. According to Khalil et al. [55], raising the pH of the solution from 2 to 8 improves the absorbance of the AgNPs produced from olive leaf extract. The absorbance falls as the pH of the solution rises more. However, the present work showed that the absorbance increased monotonically as the pH rised from 9 to 11, showing that higher alkaline pH is better for the production of AgNPs [56].

In this study, the physical conversion of silver ions to AgNPs was observed using UV-vis spectroscopy. UV-VIS spectral analysis revealed a prominent peak between 421 and 434 nm, which denoted the production of silver nanoparticles. The absorbance peak between 400 and 500 nm for indicate the production of AgNPs [57,58]. The location of the absorption band is also primarily determined by the medium's dielectric constant and the species that are surface-adsorbed. The symmetrical structure of the band indicated consistent dispersion of nanoparticles with a

spherical shape [59]. This result is consistent with that of Balavijayalakshmi and Ramalakshmi, who found that silver nanoparticles should have a spherical form [60].

The XRD confirmed that the biosynthesized AgNPS was crystalline in nature. The presence of the desirable peaks also proved that the extract includes certain organic compounds that are in charge of the stability of the resulting nanoparticles and reduction of the silver ions. The information from the XRD pattern was comparable to the results where AgNPs were found as crystalline in nature [61]. The results demonstrated that the AgNPs synthesized from Dholkolmi leaf extract composed of nanocrystals with the structure of an FCC (Face Center Cubic) pattern which was similar to the findings of Sundeep et al. [62]. The protein that was linked to the surface of the AgNPs or the biomass of the Dholkolmi leaf extract may also exhibit additional peaks in the diffraction spectrum [63]. FTIR analysis was used to classify the biomolecules in Dholkolmi leaf extract (*I. carnea*) that were in charge of the metal reduction and nanoparticle stabilization. FTIR measurements confirmed that biomolecules decreased and coated AgNPs. The bio-organic components from the *I. carnea* extract produced a potent coating or cap on the nanoparticles, according to the FTIR data. The average diameter of the particle size, was 66.2 nm in DLS method where in TEM particles sized ranged from 46.38 to 73.81 nm. The DLS measured size was slightly bigger as compared to the particle size measured from TEM micrographs because DLS method measures the hydrodynamic radius [64]. The carbon signal might have come from the coating material of the instrument's coating that was adsorbed, but the oxygen signal may have come from ambient oxygen from air. Previous research verified silver Surface Plasmon Resonance as the average absorption peak about 2.5 KeV [65,66].

AgNPs had the antifungal potentiality and our results proved that it worked well against the R. solani, the causal agent of sheath blight disease of rice. AgNPs were shown to have effectivity against rice fungi like *Bipolaris sorokiniana* and *Magnaporthe oryzae* (Jo et al., 2009; Elamawi and EL-shafey, 2013). The inhibition was 72.8 % for *Magnaporthe grisea* grown on AgNPs (20-30 nm) supplemented media at 200 mg/mL [69]. Nanoparticles are extremely reactive and enough to be effective at very less concentration with fertilizers or pesticides [70,71]. Nanoparticles are considered harmless and enhance the percent germination of seeds, vigor, plant height and reduce disease severity caused by *Fusarium oxysporium* on faba bean, barley and tomato [72]. In a prior study, 40–60 nm sized AgNPs inhibited *R. solani's* growth by 43.3–73.6 percent at a greater dose of 2 mg/mL [73]. *R. solani* showed a 67 percent inhibition with 10–20 nm AgNPs at 100 mg/mL. (Thakur et al., 2012). With *R. solani* at 50 mg/mL, the AgNPs of 5–10 nm size demon-

strated an inhibition (percentage) of 85% [75]. Complete mycelial suppression of *Aspergillus flavus* was found at 16-199mm due to AgNPs (32.7 nm) [76]. AgNPs synthesized by *A. alternata* at the concentration 100 mg/L reduced the mycelium growth of *Aspegillus flavus* and *Altenaria alternata* at a rate of 77.1% and 87.6%, respectively [77]. The AgNPs of 23 nm size caused 86 % of radial growth inhibition of *Fusarium oxysporum* at 1500 mg/mL [78]. In another study, 52.6-100% inhibition of mycelia of *Colletotrichum sp* was observed by AgNPs (4-8mm) at 100 mg/mL [41]. Whereas in the present in vitro report, 92% mycelial growth inhibition of *R. solani* found at 10:1(0.002%) ratio (pH10) with 30-90nm sizes among the other AgNPs concentration. Because of the capping of biomolecules, biogenic AgNPs have a greater strength and a smaller particle size, which may be the cause of their increased activity.

From the result of *in vivo* pot experiment, it was found that 0.002% of AgNPs was less effective against *R. solani* causing sheath blight disease of rice. But this concentration was suppressed the maximum mycelial growth in in-vitro bioassay. In the first pot experiment, maximum disease suppression was found at 0.01% AgNPs but it was not satisfactory compared to fungicide. For this reason, concentration of AgNPs was increased in the second set of pot experiment. In the second pot experiment, AgNPs (0.05%) was most effective among different AgNPs concentrations in controlling sheath blight with RLH 14.25% as compared to fungicide with RLH of 15.5%. *In vivo* net house investigations revealed that AgNPs at 50 ppm favorably influenced rice plants' fresh and dry weights while having a striking suppressive effect on the growth of leaf lesions (Soltani et al., 2015).

Nanoparticles had the capacity to defend phytopathogenic fungi. So far diverse mechanisms have been illustrated by different researcher to disclose the mode of actions of nanoparticles. By targeting cell membranes and structural components, AgNPs can denature cells. AgNPs disturbs transport mechanisms, including ion efflux, according to prior study [79]. Silver ions and silver nanoparticles have a high surface-to-volume ratio and are both known to have good antimicrobial characteristics. They can interact with the cell membrane by rupturing the cell wall structure [80]. Nanoparticles have been shown by McDonnell and Russell [81] to interact with phosphorus or sulfur. Therefore, among the ideal locations for nanoparticle binding are proteins that contain sulfur in the cell membrane or inside the cell, such as DNA [82]. The silver nanoparticles may connect with the DNA of cells due to their size and surface area. Nanoparticles with a high surface area enable them to have the best contact and interaction with the microbial cell membrane and induce the release of silver ions that work to enhance the membrane permeability in cells [83]. The thickness, make-up, peptidoglycan concentration, and lipopolysaccharide charge of the cell

wall are all potential explanations for how AgNPs affect the cell wall [84]. The most notable forms of antimicrobial action of AgNPs have been identified as adherence to microbial cells, penetration inside the cells, formation of ROS and free radicals, and regulation of microbial signal transduction pathways [85]. Overall, based on size and efficacy, AgNPs have been found as effective molecules to control diseases of different crops. Our findings, again signify the role of AgNPs to manage the plant pathogens specially to control the sheath blight disease of rice.

5. Conclusion

I. carnea (Dholkolmi) was used to illustrate a straightforward, quick, and environmentally friendly approach for producing AgNPs. The synthesis procedure was found to be efficient in terms of reaction time and the stability of the generated nanoparticles without the use of external stabilizers or reducing agents. By using a UV-visible spectrophotometer, the biosynthesized AgNPs were verified. Utilizing a DLS particle size analyzer, SEM, FESEM, TEM and Zeta potential, the size, topography, form and stability of AgNPs were determined. Invitro study revealed that t AgNPs play a significant role in mycelial growth suppression. In the pot experiments, significant disease inhibition (15.40% and 13.27% RLH) was noticed in AgNPs at 7.5ppm and 37.5ppm compared to disease control (23.12% and 24.98% RLH, respectively). In the field, the highest percentage of RLH was recorded in disease control (64.25%) and the lowest RLH was found in Amister Top (23.65% RLH) followed by 11.25ppm AgNPs (34.03% RLH), 7.5ppm AgNPs (36.80% RLH), 6ppm AgNPs (44.39% RLH) and 11.25ppm $AgNO_3$ (51.92% RLH). According our study, AgNPs had the potentiality to manage and suppress sheath blight disease. Moreover, it might be an eco-friendly management of sheath blight disease of rice.

Acknowledgement

Authors are grateful to authority of Plant Pathology Division of Bangladesh Rice Research Institute (BRRI) and Krishi Gobesona Foundation to provide funding and research facilities to conduct this experiment.

Funding

This experiment was conducted under the project titled "Sustainable management of blast, sheath blight and bacterial blight diseases of rice through Nanoparticles (Project ID:TF 71-C/20) funded by Krishi Gobeshona Foundation (KGF), Ministry of Agriculture, Bangladesh.

Conflict of interest

Authors have no any conflict of interest.

Author Contribution

AKMSI: Data curation, Software, Formal analysis, Visualization, Methodology, Resources, Validation, Writing-original draft, Writing - review & editing**. MRB:** Methodology, Data curation. **SAIN:** Data curation, Methodology, Software, Formal analysis, Visualization, Methodology, Writing-original draft, Writing - review & editing. **RA:** Methodology. **MAIK:** Methodology, Resources. **SA:** Methodology. **MARK:** Methodology, **MRI:** Methodology **MAL**: Conceptualization, Investigation, Methodology, Resources, Validation, Supervision, Writing-original draft, Writing - review & editing.

References

1. Elert E. Rice by the numbers: A good grain. Nat 2014 5147524. 2014;514: S50–S51. doi:10.1038/514s50a
2. Kasote D, Sreenivasulu N, Acuin C, Regina A. Enhancing health benefits of milled rice: current status and future perspectives. Crit Rev Food Sci Nutr. 2022;62: 8099–8119. doi:10.1080/10408398.2021.1925629
3. Mamun MA Al, Nihad SAI, Sarkar MAR, Aziz MA, Qayum MA, Ahmed R, et al. Growth and trend analysis of area, production and yield of rice: A scenario of rice security in Bangladesh. Aschonitis VG, editor. PLoS One. 2021;16: e0261128. doi:10.1371/JOURNAL.PONE.0261128
4. Akter A, Nihad SAI, Hasan MJ, Hassan L, Robin AHK, Quddus MR, et al. Evaluation of hybrid rice parental lines against bacterial blight disease and detection of resistant gene (s) by gene-specific, linked markers. J Phytopathol. 2022;170: 382–390. doi:10.1111/JPH.13087
5. Rashid MM, Nihad SAI, Khan MAI, Haque A, Ara A, Ferdous T, et al. Pathotype profiling, distribution and virulence analysis of Xanthomonas oryzae pv. oryzae causing bacterial blight disease of rice in Bangladesh. J Phytopathol. 2021;169: 438–446. doi:10.1111/jph.13000
6. Nihad S, Ara A, Rashid M, Hasan M, Khan M, Latif M. Genetic Divergence of Rice Genotypes Revealed by Bacterial Blight Disease and Morphological Traits. Bangladesh Rice J. 2021. doi:10.3329/brj.v24i1.53241
7. Nihad SAI, Manidas AC, Hasan K, Hasan MA-I, Honey O, Latif MA. Genetic variability, heritability, genetic advance and phylogenetic relationship between rice tungro virus resistant and susceptible genotypes revealed by morphological traits and SSR markers. Curr Plant Biol. 2021;25: 100194. doi:10.1016/j.cpb.2020.100194
8. Nessa B, Salam MU, Haque AHMM, Biswas JK, Latif MA, Ali MA, et al. Rice False Smut Disease at Different Flowering Times. Bangladesh Rice J. 2015;19: 28–35. doi:10.3329/BRJ.V19I2.28162
9. Latif MA, Nihad SAI, Mian MS, Akter S, Khan MAI, Ali MA. Interaction among sheath diseases complex of rice and ribosomal DNA analysis for the differentiation of Rhizoctonia solani, R. oryzae and R. oryzae-sativae. Plant Stress. 2022;5: 100100. doi:https://doi.org/10.1016/j.stress.2022.100100

10. Savary S, Willocquet L, Elazegui FA, Teng PS, Van Du P, Zhu D, et al. Rice Pest Constraints in Tropical Asia: Characterization of Injury Profiles in Relation to Production Situations. https://doi.org/101094/PDIS2000843341. 2007;84: 341–356. doi:10.1094/PDIS.2000.84.3.341
11. Nagarajkumar M, Bhaskaran R, Velazhahan R. Involvement of secondary metabolites and extracellular lytic enzymes produced by Pseudomonas fluorescens in inhibition of Rhizoctonia solani, the rice sheath blight pathogen. Microbiol Res. 2004;159: 73–81. doi:10.1016/J.MICRES.2004.01.005
12. Wang L, Liu LM, Hou YX, Li L, Huang SW. Pathotypic and genetic diversity in the population of Rhizoctonia solani AG1-IA causing rice sheath blight in China. Plant Pathol. 2015;64: 718–728. doi:10.1111/PPA.12299
13. Boukaew S, Klinmanee C, Prasertsan P. Potential for the integration of biological and chemical control of sheath blight disease caused by Rhizoctonia solani on rice. World J Microbiol Biotechnol. 2013;29: 1885–1893. doi:10.1007/S11274-013-1353-X
14. Panda N, Heinrichs EA, Hibino H. Resistance of the rice variety Utri Rajapan to ragged stunt and tungro viruses. Crop Prot. 1984. doi:10.1016/0261-2194(84)90030-9
15. S.S K, N P. EFFECT OF FOLIAR SPRAY OF CERTAIN FUNGICIDES ON THE CONTROL OF SHEATH BLIGHT DISEASE OF RICE. Madras Agric J. 1984;71: 111–114. doi:10.29321/MAJ.10.A02446
16. Zhang CQ, Liu YH, Ma XY, Feng Z, Ma ZH. Characterization of sensitivity of Rhizoctonia solani, causing rice sheath blight, to mepronil and boscalid. Crop Prot. 2009;28: 381–386. doi:10.1016/J.CROPRO.2008.12.004
17. Islam R, Nihad SAI, Obaidullah AJM, Mamun A Al, Moniruzzaman M, Hasan S. Efficacy of biological, chemical and cultural practices for the management of foot and root rot disease of black cumin. Biocatal Agric Biotechnol. 2021. doi:10.1016/j.bcab.2021.102193
18. Dutta P, Kumari A, Mahanta M, Biswas KK, Dudkiewicz A, Thakuria D, et al. Advances in Nanotechnology as a Potential Alternative for Plant Viral Disease Management. Front Microbiol. 2022;13: 935193. doi:10.3389/FMICB.2022.935193
19. Joudeh N, Linke D. Nanoparticle classification, physicochemical properties, characterization, and applications: a comprehensive review for biologists. J Nanobiotechnology 2022 201. 2022;20: 1–29. doi:10.1186/S12951-022-01477-8
20. Altammar KA. A review on nanoparticles: characteristics, synthesis, applications, and

challenges. Front Microbiol. 2023;14. doi:10.3389/FMICB.2023.1155622

21. Chakrabarti M, Kiseleva R, Vertegel A, Ray SK. Carbon Nanomaterials for Drug Delivery and Cancer Therapy. J Nanosci Nanotechnol. 2015;15: 5501–5511. doi:10.1166/JNN.2015.10614

22. Amini A, Yang C, Cheng C, Naebe M, Xiang Y. Nanoscale variation in energy dissipation in austenitic shape memory alloys in ultimate loading cycles. http://dx.doi.org/101177/1045389X14560365. 2014;26: 2411–2417. doi:10.1177/1045389X14560365

23. Khatami M, Pourseyedi S, Khatami M, Hamidi H, Zaeifi M, Soltani L. Synthesis of silver nanoparticles using seed exudates of sinapis arvensis as a novel bioresource, and evaluation of their antifungal activity. Bioresour Bioprocess. 2015;2: 1–7. doi:10.1186/S40643-015-0043-Y/FIGURES/6

24. Mekuye B, Abera B. Nanomaterials: An overview of synthesis, classification, characterization, and applications. Nano Sel. 2023;4: 486–501. doi:10.1002/NANO.202300038

25. Worrall EA, Hamid A, Mody KT, Mitter N, Pappu HR. Nanotechnology for Plant Disease Management. Agron 2018, Vol 8, Page 285. 2018;8: 285. doi:10.3390/AGRONOMY8120285

26. Kathiraven T, Sundaramanickam A, Shanmugam N, Balasubramanian T. Green synthesis of silver nanoparticles using marine algae Caulerpa racemosa and their antibacterial activity against some human pathogens. Appl Nanosci. 2015;5: 499–504. doi:10.1007/S13204-014-0341-2/FIGURES/7

27. Parisi C, Vigani M, Rodríguez-Cerezo E. Agricultural Nanotechnologies: What are the current possibilities? Nano Today. 2015;10: 124–127. doi:10.1016/J.NANTOD.2014.09.009

28. Anjali C, Sharma Y, Mukherjee A, Chandrasekaran N. Neem oil (Azadirachta indica) nanoemulsion--a potent larvicidal agent against Culex quinquefasciatus. Pest Manag Sci. 2012;68: 158–163. doi:10.1002/PS.2233

29. Milani N, McLaughlin MJ, Stacey SP, Kirby JK, Hettiarachchi GM, Beak DG, et al. Dissolution kinetics of macronutrient fertilizers coated with manufactured zinc oxide nanoparticles. J Agric Food Chem. 2012;60: 3991–3998. doi:10.1021/JF205191Y

30. Ajith MP, Aswathi M, Priyadarshini E, Rajamani P. Recent innovations of nanotechnology in water treatment: A comprehensive review. Bioresour Technol.

2021;342: 126000. doi:10.1016/J.BIORTECH.2021.126000
31. Vamvakaki V, Chaniotakis NA. Pesticide detection with a liposome-based nano-biosensor. Biosens Bioelectron. 2007;22: 2848–2853. doi:10.1016/J.BIOS.2006.11.024
32. Torney F, Trewyn BG, Lin VSY, Wang K. Mesoporous silica nanoparticles deliver DNA and chemicals into plants. Nat Nanotechnol. 2007;2: 295–300. doi:10.1038/NNANO.2007.108
33. Njagi EC, Huang H, Stafford L, Genuino H, Galindo HM, Collins JB, et al. Biosynthesis of iron and silver nanoparticles at room temperature using aqueous sorghum bran extracts. Langmuir. 2011;27: 264–271. doi:10.1021/LA103190N/ASSET/IMAGES/MEDIUM/LA-2010-03190N_0012.GIF
34. Lee HJ, Song JY, Kim BS. Biological synthesis of copper nanoparticles using Magnolia kobus leaf extract and their antibacterial activity. J Chem Technol Biotechnol. 2013;88: 1971–1977. doi:10.1002/JCTB.4052
35. Ghaedi M, Yousefinejad M, Safarpoor M, Khafri HZ, Purkait MK. Rosmarinus officinalis leaf extract mediated green synthesis of silver nanoparticles and investigation of its antimicrobial properties. J Ind Eng Chem. 2015;31: 167–172. doi:10.1016/J.JIEC.2015.06.020
36. Villamizar-Gallardo R, Cruz JFO, Ortíz OO. Fungicidal effect of silver nanoparticles on toxigenic fungi in cocoa. Pesqui Agropecu Bras. 2016;51. doi:10.1590/S0100-204X2016001200003
37. Anees Ahmad S, Sachi Das S, Khatoon A, Tahir Ansari M, Afzal M, Saquib Hasnain M, et al. Bactericidal activity of silver nanoparticles: A mechanistic review. Materials Science for Energy Technologies. 2020. doi:10.1016/j.mset.2020.09.002
38. Pal S, Tak YK, Song JM. Does the antibacterial activity of silver nanoparticles depend on the shape of the nanoparticle? A study of the Gram-negative bacterium Escherichia coli. Appl Environ Microbiol. 2007;73: 1712–1720. doi:10.1128/AEM.02218-06
39. Dibrov P, Dzioba J, Gosink KK, Häse CC. Chemiosmotic Mechanism of Antimicrobial Activity of Ag+ in Vibrio cholerae. Antimicrob Agents Chemother. 2002;46: 2668. doi:10.1128/AAC.46.8.2668-2670.2002
40. Braydich-Stolle L, Hussain S, Schlager JJ, Hofmann MC. In Vitro Cytotoxicity of Nanoparticles in Mammalian Germline Stem Cells. Toxicol Sci. 2005;88: 412. doi:10.1093/TOXSCI/KFI256
41. Lamsal K, Kim SW, Jung JH, Kim YS, Kim KS, Lee YS. Application of silver

nanoparticles for the control of colletotrichum species in vitro and pepper anthracnose disease in field. Mycobiology. 2011;39: 194–199. doi:10.5941/MYCO.2011.39.3.194

42. Li L, Pan H, Deng L, Qian G, Wang Z, Li W, et al. The antifungal activity and mechanism of silver nanoparticles against four pathogens causing kiwifruit post-harvest rot. Front Microbiol. 2022;13. doi:10.3389/FMICB.2022.988633/FULL

43. Kumar V, Yadav SK. Plant-mediated synthesis of silver and gold nanoparticles and their applications. J Chem Technol Biotechnol. 2009;84: 151–157. doi:10.1002/JCTB.2023

44. Zhang M, Zhang K, De Gusseme B, Verstraete W, Field R. The antibacterial and anti-biofouling performance of biogenic silver nanoparticles by Lactobacillus fermentum. Biofouling. 2014;30: 347–357. doi:10.1080/08927014.2013.873419

45. Huang J, Zhan G, Zheng B, Sun D, Lu F, Lin Y, et al. Biogenic silver nanoparticles by Cacumen Platycladi extract: Synthesis, formation mechanism, and antibacterial activity. Ind Eng Chem Res. 2011;50: 9095–9106. doi:10.1021/IE200858Y/SUPPL_FILE/IE200858Y_SI_001.PDF

46. Hebbalalu D, Lalley J, Nadagouda MN, Varma RS. Greener techniques for the synthesis of silver nanoparticles using plant extracts, enzymes, bacteria, biodegradable polymers, and microwaves. ACS Sustain Chem Eng. 2013;1: 703–712. doi:10.1021/SC4000362/ASSET/IMAGES/MEDIUM/SC-2013-000362_0002.GIF

47. Usha Rani P, Rajasekharreddy P. Green synthesis of silver-protein (core–shell) nanoparticles using Piper betle L. leaf extract and its ecotoxicological studies on Daphnia magna. Colloids Surfaces A Physicochem Eng Asp. 2011;389: 188–194. doi:10.1016/J.COLSURFA.2011.08.028

48. Vanlalveni C, Lallianrawna S, Biswas A, Selvaraj M, Changmai B, Rokhum SL. Green synthesis of silver nanoparticles using plant extracts and their antimicrobial activities: a review of recent literature. RSC Adv. 2021;11: 2804. doi:10.1039/D0RA09941D

49. Dhand V, Soumya L, Bharadwaj S, Chakra S, Bhatt D, Sreedhar B. Green synthesis of silver nanoparticles using Coffea arabica seed extract and its antibacterial activity. Mater Sci Eng C Mater Biol Appl. 2016;58: 36–43. doi:10.1016/J.MSEC.2015.08.018

50. Ali K, Ahmed B, Dwivedi S, Saquib Q, Al-Khedhairy AA, Musarrat J. Microwave Accelerated Green Synthesis of Stable Silver Nanoparticles with Eucalyptus globulus Leaf Extract and Their Antibacterial and Antibiofilm Activity on Clinical Isolates. PLoS One. 2015;10: e0131178. doi:10.1371/JOURNAL.PONE.0131178

51. De Balogh KKIM, Dimande AP, Van Der Lugt JJ, Molyneux RJ, Naudé TW, Welman

WG. A lysosomal storage disease induced by Ipomoea carnea in goats in Mozambique. J Vet Diagn Invest. 1999;11: 266–273. doi:10.1177/104063879901100310

52. Verma A, Mehata MS. Controllable synthesis of silver nanoparticles using Neem leaves and their antimicrobial activity. J Radiat Res Appl Sci. 2016;9: 109–115. doi:10.1016/J.JRRAS.2015.11.001

53. Alqadi MK, Abo Noqtah OA, Alzoubi FY, Alzouby J, Aljarrah K. PH effect on the aggregation of silver nanoparticles synthesized by chemical reduction. Mater Sci Pol. 2014;32: 107–111. doi:10.2478/S13536-013-0166-9/METRICS

54. Akhatova F, Konnova S, Kryuchkova M, Batasheva S, Mazurova K, Vikulina A, et al. Comparative Characterization of Iron and Silver Nanoparticles: Extract-Stabilized and Classical Synthesis Methods. Int J Mol Sci. 2023;24: 9274. doi:10.3390/IJMS24119274/S1

55. Khalil MMH, Ismail EH, El-Baghdady KZ, Mohamed D. Green synthesis of silver nanoparticles using olive leaf extract and its antibacterial activity. Arab J Chem. 2014;7: 1131–1139. doi:10.1016/J.ARABJC.2013.04.007

56. Vanaja M, Rajeshkumar S, Paulkumar K, Gnanajobitha G, Malarkodi C, Annadurai G. Kinetic study on green synthesis of silver nanoparticles using Coleus aromaticus leaf extract. Adv Appl Sci Res. 2013.

57. Gene RP, Jain P, Singh PK, Kapoor R, Khanna A. Understanding Host-Pathogen Interactions with Expression Profiling of NILs Carrying Rice-Blast. 2017;8: 1–20. doi:10.3389/fpls.2017.00093

58. Ajayi E, Afolayan A. Green synthesis, characterization and biological activities of silver nanoparticles from alkalinized Cymbopogon citratus Stapf. Adv Nat Sci Nanosci Nanotechnol. 2017;8: 015017. doi:10.1088/2043-6254/AA5CF7

59. Sreelakshmy V. Green Synthesis of Silver Nanoparticles from Glycyrrhiza glabra Root Extract for the Treatment of Gastric Ulcer. 2016. doi:10.4172/2329-6631.1000152

60. Balavijayalakshmi J, Ramalakshmi V. Carica papaya peel mediated synthesis of silver nanoparticles and its antibacterial activity against human pathogens. J Appl Res Technol. 2017;15: 413–422. doi:10.1016/J.JART.2017.03.010

61. Pirtarighat S, Ghannadnia M, Baghshahi S. Green synthesis of silver nanoparticles using the plant extract of Salvia spinosa grown in vitro and their antibacterial activity assessment. J Nanostructure Chem. 2019;9: 1–9. doi:10.1007/S40097-018-0291-4/FIGURES/6

62. Sundeep D, Vijaya Kumar T, Rao PSS, Ravikumar RVSSN, Gopala Krishna A. Green synthesis and characterization of Ag nanoparticles from Mangifera indica leaves for dental restoration and antibacterial applications. Prog Biomater. 2017;6: 57. doi:10.1007/S40204-017-0067-9
63. Shankar SS, Rai A, Ahmad A, Sastry M. Rapid synthesis of Au, Ag, and bimetallic Au core–Ag shell nanoparticles using Neem (Azadirachta indica) leaf broth. J Colloid Interface Sci. 2004;275: 496–502. doi:10.1016/J.JCIS.2004.03.003
64. Huang NM, Lim HN, Radiman S, Khiew PS, Chiu WS, Hashim R, et al. Sucrose ester micellar-mediated synthesis of Ag nanoparticles and the antibacterial properties. Colloids Surfaces A Physicochem Eng Asp. 2010;353: 69–76. doi:10.1016/J.COLSURFA.2009.10.023
65. Rautela A, Rani J, Debnath (Das) M. Green synthesis of silver nanoparticles from Tectona grandis seeds extract: characterization and mechanism of antimicrobial action on different microorganisms. J Anal Sci Technol. 2019;10: 1–10. doi:10.1186/S40543-018-0163-Z/FIGURES/14
66. El-Sadony MTM. Biosynthesis, Optimization and Characterization of Silver Nanoparticles Using a Soil Isolate of Bacillus pseudomycoides MT32 and their Antifungal Activity Against some Pathogenic Fungi. Biosynthesis, Optim.
67. Jo YK, Kim BH, Jung G. Antifungal activity of silver ions and nanoparticles on phytopathogenic fungi. Plant Dis. 2009;93. doi:10.1094/PDIS-93-10-1037
68. ELAMAWI RMA, EL-SHAFEY RA. INHIBITION EFFECTS OF SILVER NANOPARTICLES AGAINST RICE BLAST DISEASE CAUSED BY MAGNAPORTHE GRISEA. Egypt J Agric Res. 2013;91. doi:10.21608/ejar.2013.165104
69. El-Esawi MA, Elansary HO, El-Shanhorey NA, Abdel-Hamid AME, Ali HM, Elshikh MS. Salicylic acid-regulated antioxidant mechanisms and gene expression enhance rosemary performance under saline conditions. Front Physiol. 2017;8: 716. doi:10.3389/FPHYS.2017.00716/FULL
70. Batsmanova LM, Gonchar LM, Taran NY, Okanenko AA. Using a colloidal solution of metal nanoparticles as micronutrient fertiliser for cereals. Proc 2nd Int Conf - Nanomater Appl Prop. 2013;2.
71. Elamawi R, Bassiouni S, Elkhoby W, Zayed B. EFFECT OF ZINC OXIDE NANOPARTICLES ON BROWN SPOT DISEASE AND RICE PRODUCTIVITY UNDER SALINE SOIL. J Plant Prot Pathol. 2016;7. doi:10.21608/jppp.2016.50137

72. Elamawi R, Al-Harbi R. EFFECT OF BIOSYNTHESIZED SILVER NANOPARTICLES ON Fusarium oxysporum FUNGUS THE CAUSE OF SEED ROT DISEASE OF FABA BEAN, TOMATO AND BARLEY. J Plant Prot Pathol. 2014;5. doi:10.21608/jppp.2014.87901
73. Elgorban AM, El-Samawaty AERM, Yassin MA, Sayed SR, Adil SF, Elhindi KM, et al. Antifungal silver nanoparticles: synthesis, characterization and biological evaluation. Biotechnol Biotechnol Equip. 2016;30: 56–62. doi:10.1080/13102818.2015.1106339
74. Thakur RN. An in vitro study of the antifungal activity of silver/chitosan nanoformulations against important seed borne pathogens. Available: https://www.academia.edu/15077263/An_in_vitro_study_of_the_antifungal_activity_of_silver_chitosan_nanoformulations_against_important_seed_borne_pathogens
75. M. S, M. K, G.H. SB. STREPTOMYCES SOMALIENSIS MEDIATED GREEN SYNTHESIS OF SILVER NANOPARTICLES. Nanomed. J. NANOMEDICINE JOURNAL; 2015. pp. 217–222. doi:10.7508/nmj
76. Hashem AH, Saied E, Amin BH, Alotibi FO, Al-Askar AA, Arishi AA, et al. Antifungal Activity of Biosynthesized Silver Nanoparticles (AgNPs) against Aspergilli Causing Aspergillosis: Ultrastructure Study. J Funct Biomater. 2022;13. doi:10.3390/JFB13040242/S1
77. Al-Zaban MI, Mahmoud MA, Alharbi MA. A polyphasic approach to the characterization of potential silver-nanoparticles–producing and non-producing isolates of Alternaria species and antifungal activity against mycotoxigenic fungi. Biotechnol Biotechnol Equip. 2021;35: 341–353. doi:10.1080/13102818.2021.1875876
78. Surega R, Ramakrishnan S, Anita B, Gunasekaran K, Nakkeeran S. Antifungal effect of plant mediated silver nanoparticles against Fusarium oxysporum f sp lycopersici. Int J Trop Agric. 2015;33: 157–160.
79. Morones JR, Elechiguerra JL, Camacho A, Holt K, Kouri JB, Ramírez JT, et al. The bactericidal effect of silver nanoparticles. Nanotechnology. 2005;16. doi:10.1088/0957-4484/16/10/059
80. Yin IX, Zhang J, Zhao IS, Mei ML, Li Q, Chu CH. The Antibacterial Mechanism of Silver Nanoparticles and Its Application in Dentistry. Int J Nanomedicine. 2020;15: 2555. doi:10.2147/IJN.S246764
81. Bhuvaneswari T, Thiyagarajan M, Geetha N, Venkatachalam P. Bioactive compound loaded stable silver nanoparticle synthesis from microwave irradiated aqueous

extracellular leaf extracts of Naringi crenulata and its wound healing activity in experimental rat model. Acta Trop. 2014;135: 55–61. doi:10.1016/J.ACTATROPICA.2014.03.009
82. Velusamy P, Kumar GV, Jeyanthi V, Das J, Pachaiappan R. Bio-Inspired Green Nanoparticles: Synthesis, Mechanism, and Antibacterial Application. Toxicol Res. 2016;32: 95. doi:10.5487/TR.2016.32.2.095
83. Khatua A, Prasad A, Priyadarshini E, Virmani I, Ghosh llora, Paul B, et al. CTAB-PLGA Curcumin Nanoparticles: Preparation, Biophysical Characterization and Their Enhanced Antifungal Activity against Phytopathogenic Fungus Pythium ultimum. ChemistrySelect. 2020;5: 10574–10580. doi:10.1002/SLCT.202002158
84. Godoy-Gallardo M, Eckhard U, Delgado LM, de Roo Puente YJD, Hoyos-Nogués M, Gil FJ, et al. Antibacterial approaches in tissue engineering using metal ions and nanoparticles: From mechanisms to applications. Bioact Mater. 2021;6: 4470–4490. doi:10.1016/J.BIOACTMAT.2021.04.033
85. Dakal TC, Kumar A, Majumdar RS, Yadav V. Mechanistic Basis of Antimicrobial Actions of Silver Nanoparticles. Front Microbiol. 2016;7: 1831. doi:10.3389/FMICB.2016.01831

Figure Legends

Figure 1. Color change after AgNPs formation (a-d) and UV-Vis spectra showing absorbance with different concentration and pH of plant extract (e-h).

Figure 2. X-Ray Diffraction pattern (a), FTIR spectrum (b), particle size diameter (c) and Zeta potential (d) of AgNPs

Figure 3. SEM (a), FESEM (b), EDX spectra (c), and TEM (d) images of biosynthesized AgNPs.

Figure 4. Efficacy of synthesized AgNPs against *R. solani* mycelium growth inhibition in in vitro condition. Here, (a) Fungicide, (b) AgNO3, (c) AgNPs, (d) Disease control (*R. solani*), (e) Leaf extract and (f) Water (g) effect of dholkolmi leaf extract mediated AgNPs on mycelial growth of *R. solani*. Here, Ag= AgNO3, D = Dholkolmi leaves extract.

Figure 5. Hourly efficacy of AgNPs at different concentration and pH to control the mycelium growth of *R. solani* at invitro condition.

Figure 6. Efficacy of AgNPs against sheath blight disease of rice in pot (a-b) and field experiments (c).

Figure 7. Sheath blight disease suppression due to application of AgNPs application. Here, (a) disease control in pot (b) AgNPs treated pot (c) disease control in field (d) AgNPs treated plant in field. Red mark tick indicated the sheath blight disease symptoms in the disease control plant.

Figure 1

Click here to access/download;Figure;Figure 1.tif

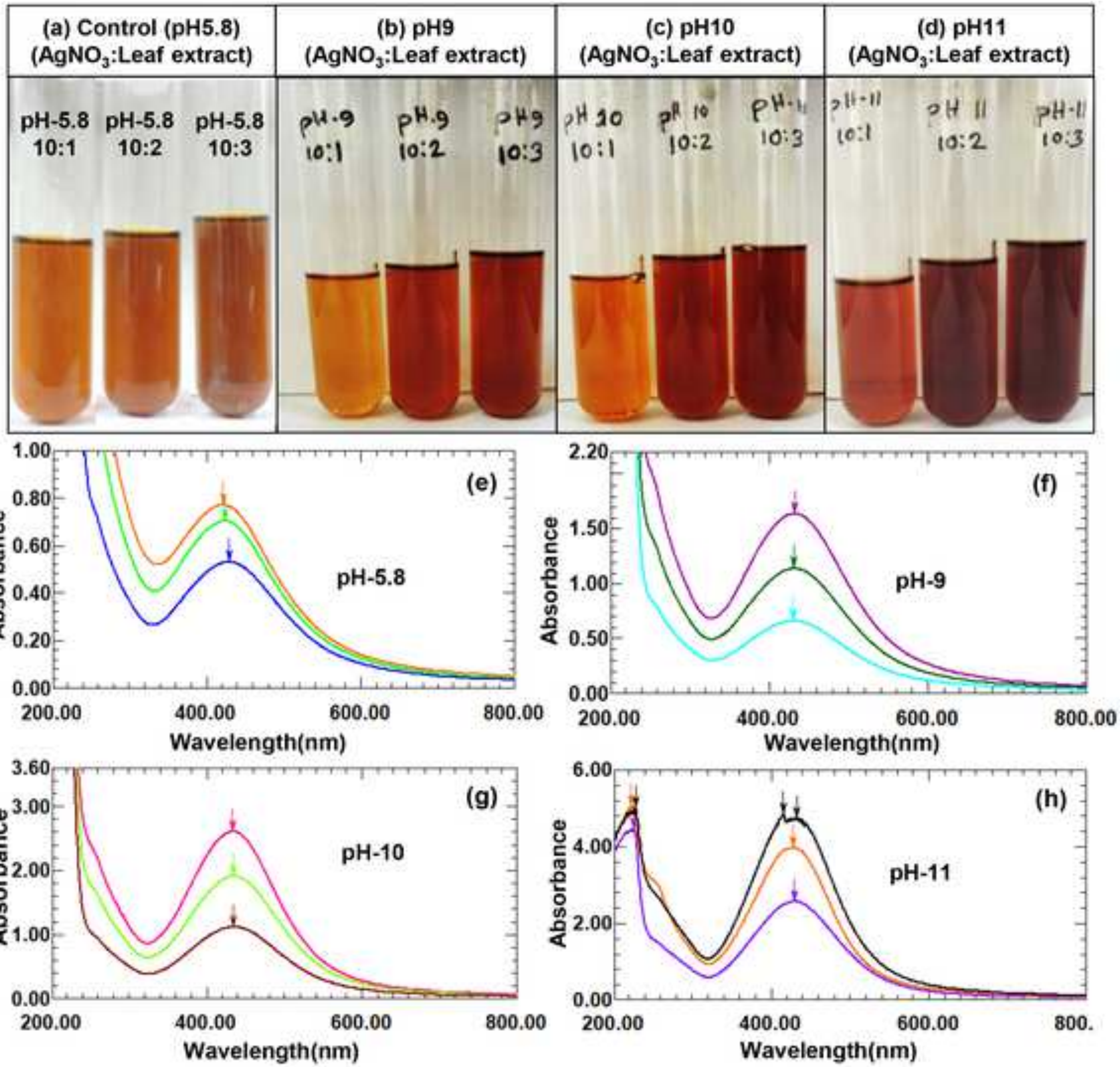

Figure 2

Click here to access/download;Figure;Figure 2.tif

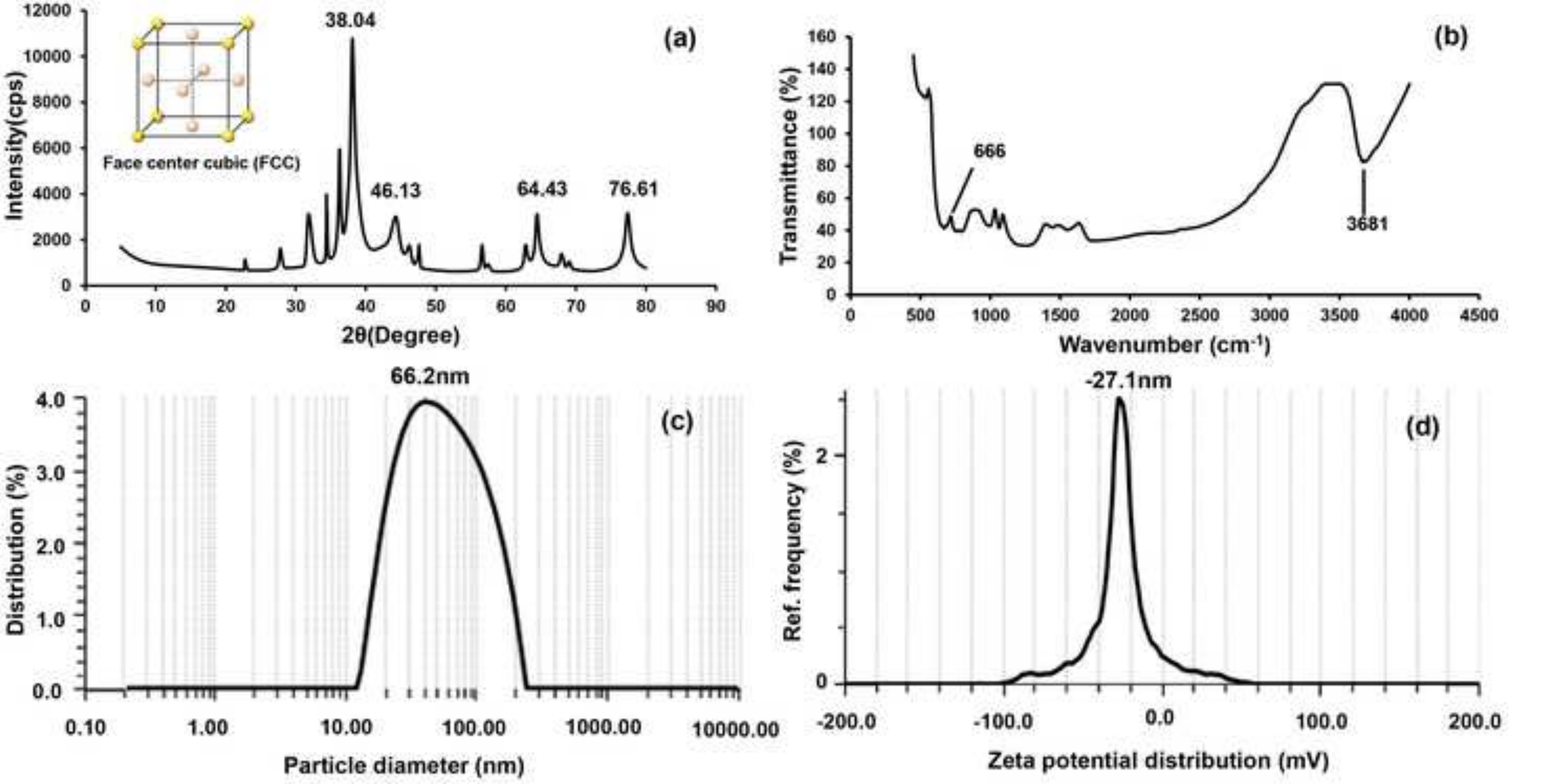

Figure 3

Click here to access/download;Figure;Figure 3.tif

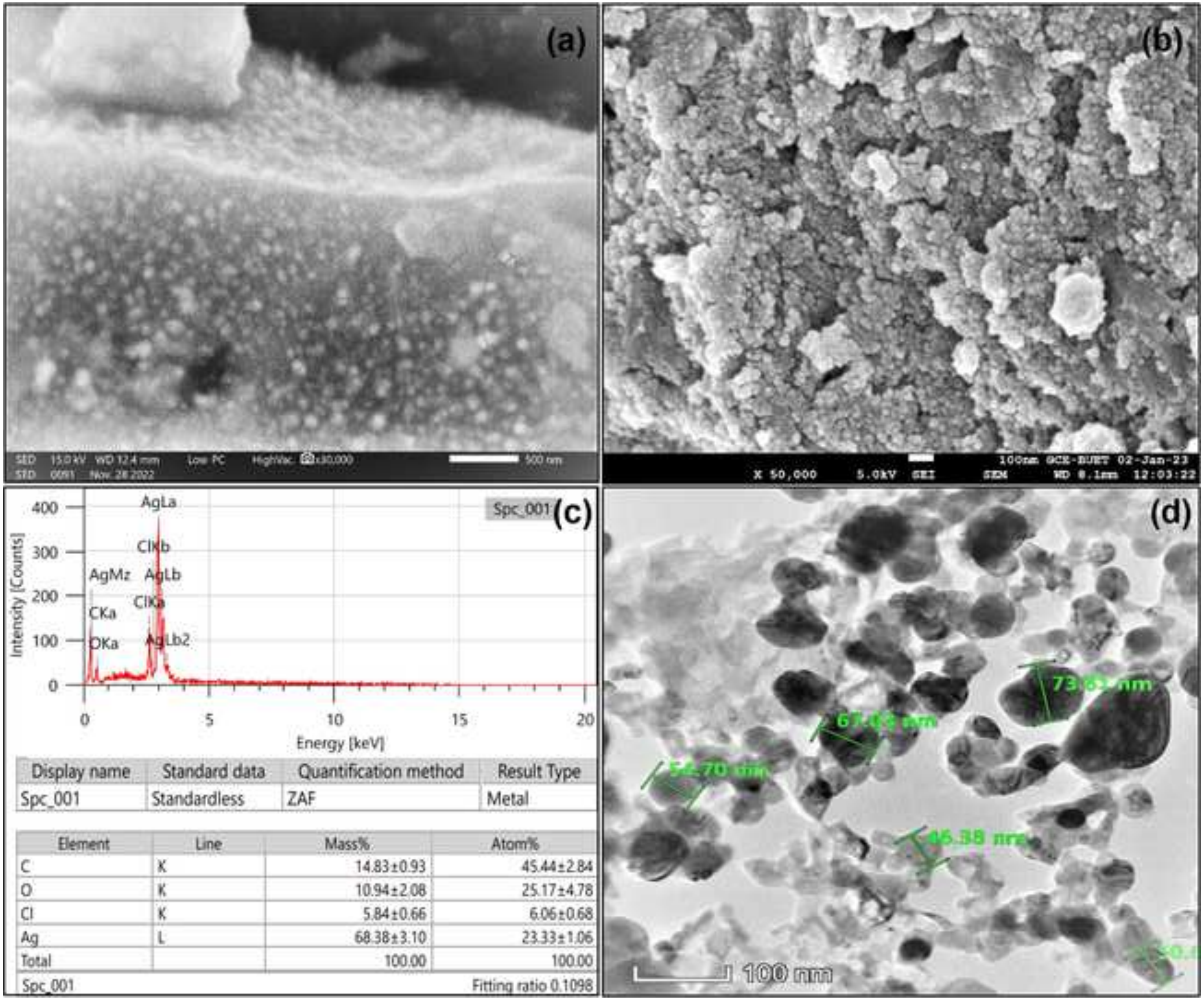

Figure 4

Click here to access/download;Figure;Figure 4.tif

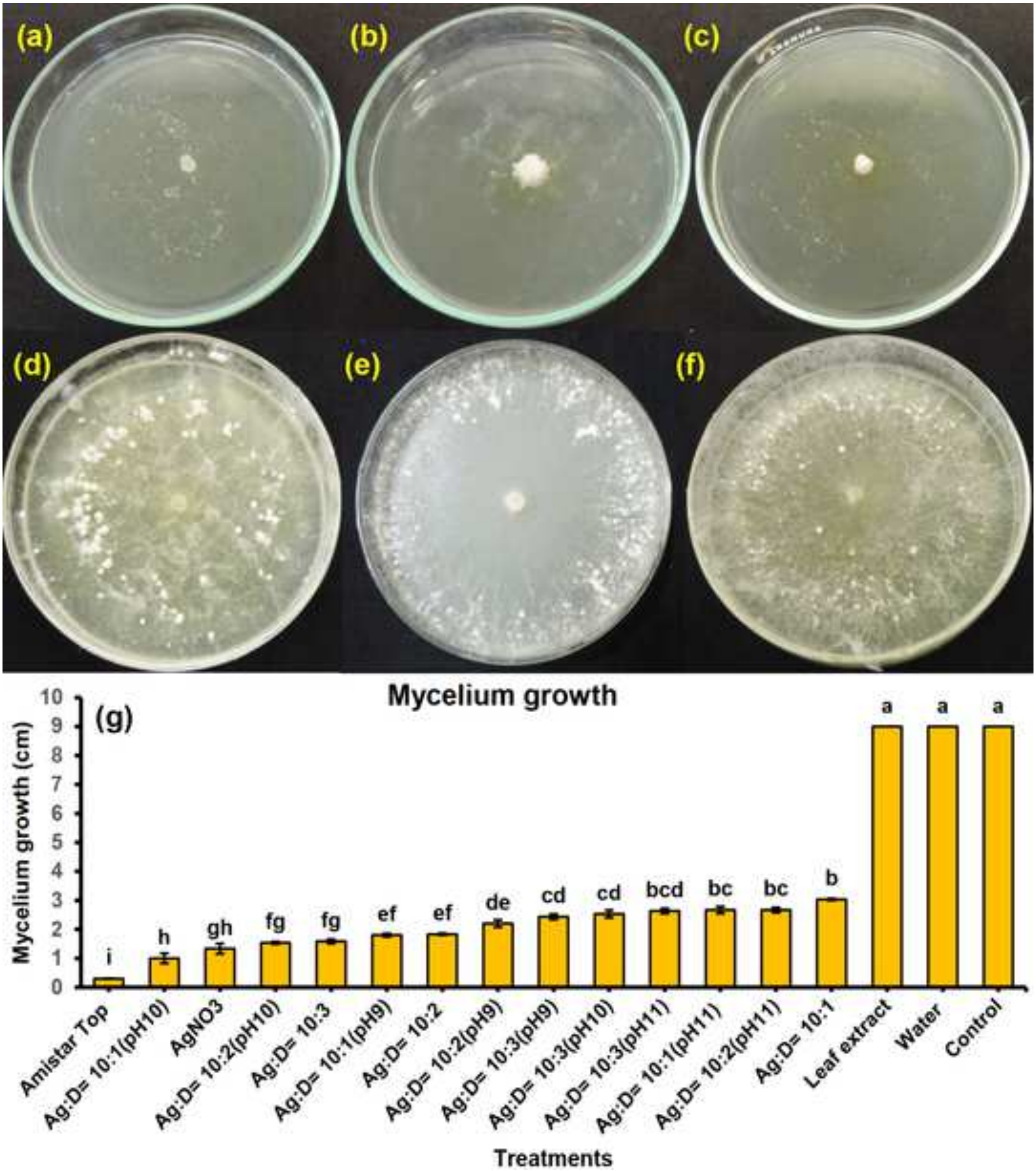

Figure 5

Click here to access/download;Figure;Figure 5.tif

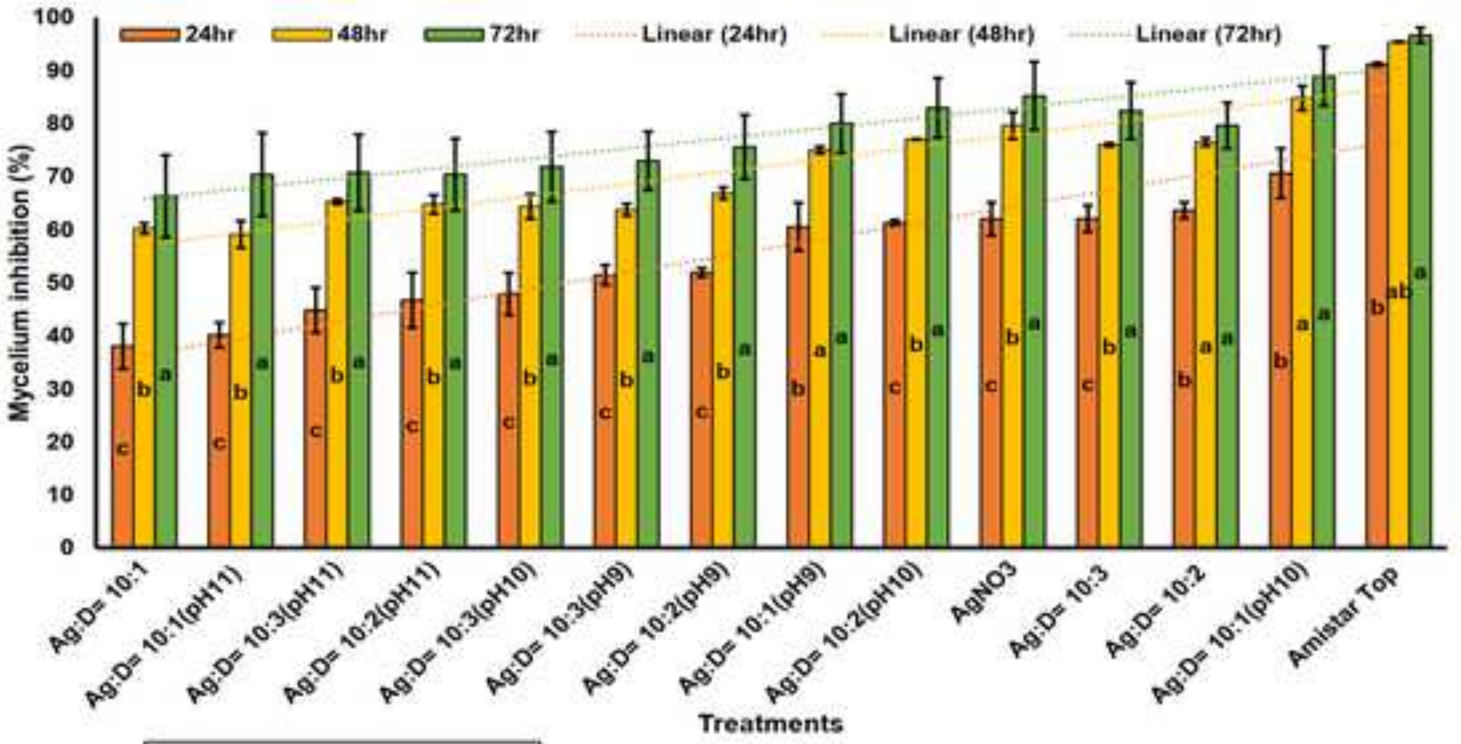

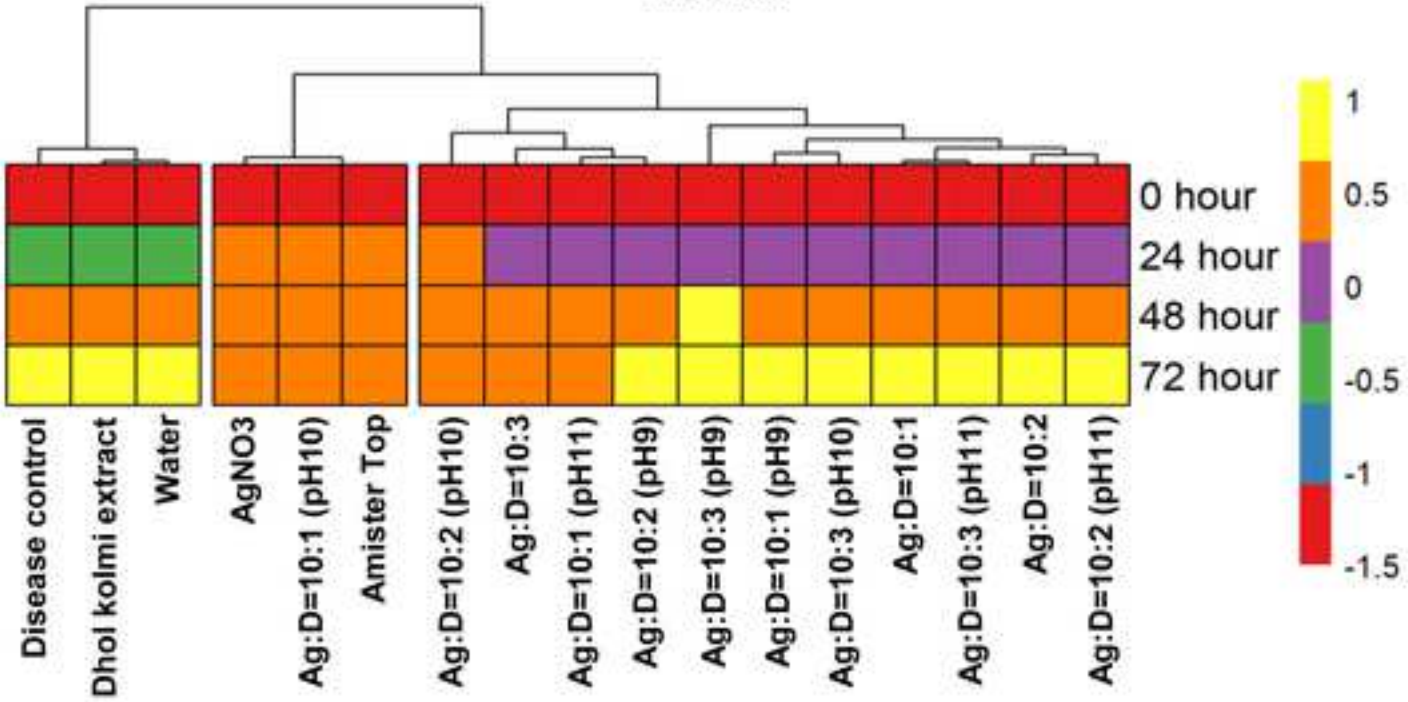

Figure 6

Click here to access/download;Figure;Figure 6.tif

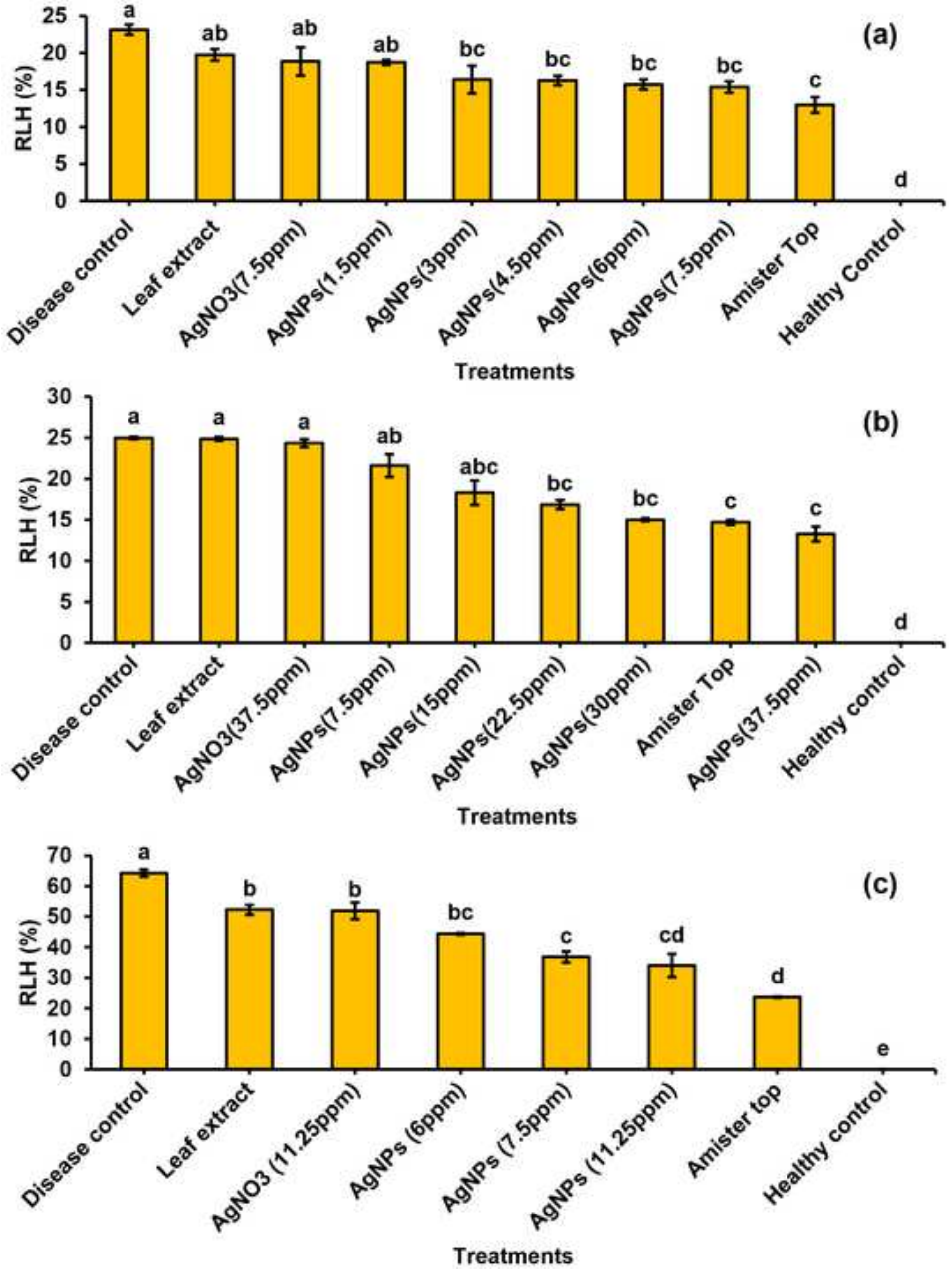

Figure 7

Click here to access/download;Figure;Figure 7.tif

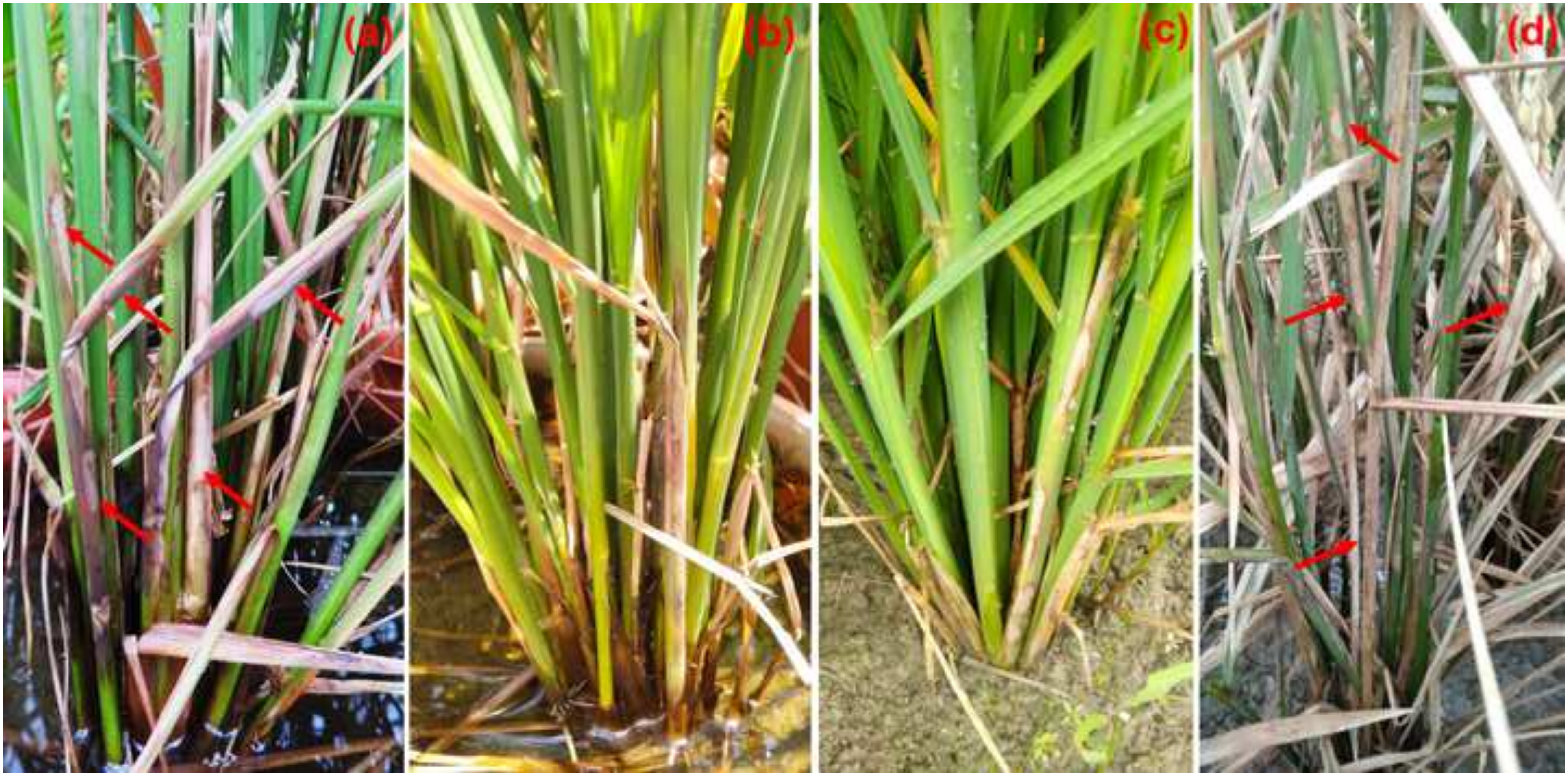

Supplement: Attachment — Submitted filename: PONE-D-23-39382_reviewer.pdf [file pone.0304817.s002.pdf]
