# Supplementary material for: Green synthesis and characterization of silver nanoparticles and its efficacy against Rhizoctonia solani, a fungus causing sheath blight disease in rice
Source: PLoS One. 2024 Jun 18;19(6):e0304817. doi: 10.1371/journal.pone.0304817 (PMC11185457; doi:10.1371/journal.pone.0304817)
Supplement: Attachment — Submitted filename: COMMENTS.docx [file pone.0304817.s003.docx]

COMMENTS

The article lacks the innovation, since similar work has been published before, please follow this link: <https://doi.org/10.1049/iet-nbt.2015.0121>

The English language of the manuscript is very poor. It must be improved (please see carefully the attached revised version). The manuscript is difficult to publish before the editing of English language.

The Abstract

The abstract is too long; it should be shortened and the abbreviations should not mention in the abstract. For example, the genera of organisms should be written in full.

AgNO3 correct to AgNO_3_ throughout the manuscript.

In Materials and Methods:

The paragraph 2.2. is confusing and must be modified for understanding.

The authors did not explain the characteristics of soil used in pot experiment.

The paragraph 2.6. is confusing and must be modified for understanding.

Line 303: What are the authors means by the 3-day old mycelial blocks *R. solani* were placed in the center of the plants?

Dholkolimi or dholkolimi, please unify throughout the manuscript.

Amister or amister,

In vivo or in vitro should be written in italic.

The color or colour, please unify throughout the manuscript.

Min. or minutes, please unify throughout the manuscript.

The author must follow the style of the journal during writing particularly the references list.
