# Supplementary material for: Green synthesis and characterization of silver nanoparticles and its efficacy against Rhizoctonia solani, a fungus causing sheath blight disease in rice
Source: PLoS One. 2024 Jun 18;19(6):e0304817. doi: 10.1371/journal.pone.0304817 (PMC11185457; doi:10.1371/journal.pone.0304817)
Supplement: Attachment — Submitted filename: Response to editor and reviewers comments.docx [file pone.0304817.s004.docx]

**Response to Editor and reviewer’s comments**

**Journal Requirements:**

**When submitting your revision, we need you to address these additional requirements.**

**Comment 1**: Please ensure that your manuscript meets PLOS ONE's style requirements, including those for file naming. The PLOS ONE style templates can be found at 
<https://journals.plos.org/plosone/s/file?id=wjVg/PLOSOne_formatting_sample_main_body.pdf> and 
<https://journals.plos.org/plosone/s/file?id=ba62/PLOSOne_formatting_sample_title_authors_affiliations.pdf>

Response: We checked and tried to arrange the manuscript according to PLOS ONE journal format.

**Comment 2**.  We suggest you thoroughly copyedit your manuscript for language usage, spelling, and grammar.

**Response:** We tried to revise the language, spelling and grammer of our manuscript. The manuscript has been edited by Dr. Mohammad Abdul Latif, Director, Admin and Common Service and Former Head and Chief Scientific Officer of the Plant Pathology Division of Bangladesh Rice Research Institute (BRRI), Gazipur, Bangladesh.

**Comment 3**: Thank you for stating the following financial disclosure: 
"This experiment was conducted under the project titled “Sustainable management of blast, sheath blight and bacterial blight diseases of rice through Nanoparticles (Project ID:TF 71-C/20) funded by Krishi Gobeshona Foundation (KGF), Ministry of Agriculture, Bangladesh."
 
Please state what role the funders took in the study.  If the funders had no role, please state: ""The funders had no role in study design, data collection and analysis, decision to publish, or preparation of the manuscript.""

Response: We already included the statement "The funders had no role in study design, data collection and analysis, decision to publish, or preparation of the manuscript” in the funding section.

**Comment 4**. Thank you for stating the following in the Acknowledgments Section of your manuscript: 
"Authors are grateful to authority of Plant Pathology Division of Bangladesh Rice Research Institute (BRRI) and Krishi Gobesona Foundation to provide funding and research facilities to conduct this experiment."

We note that you have provided funding information that is not currently declared in your Funding Statement. However, funding information should not appear in the Acknowledgments section or other areas of your manuscript. We will only publish funding information present in the Funding Statement section of the online submission form. 
Please remove any funding-related text from the manuscript and let us know how you would like to update your Funding Statement. Currently, your Funding Statement reads as follows: 
"This experiment was conducted under the project titled “Sustainable management of blast, sheath blight and bacterial blight diseases of rice through Nanoparticles (Project ID:TF 71-C/20) funded by Krishi Gobeshona Foundation (KGF), Ministry of Agriculture, Bangladesh."

Please include your amended statements within your cover letter; we will change the online submission form on your behalf.

Response:  We removed the funding information from the Acknowledgement section and updated the funding statement in the Funding section.

**Comment 5.** We note that your Data Availability Statement is currently as follows: All relevant data are within the manuscript and its Supporting Information files.

Please confirm at this time whether or not your submission contains all raw data required to replicate the results of your study. Authors must share the “minimal data set” for their submission. PLOS defines the minimal data set to consist of the data required to replicate all study findings reported in the article, as well as related metadata and methods (<https://journals.plos.org/plosone/s/data-availability#loc-minimal-data-set-definition>).

For example, authors should submit the following data:

- The values behind the means, standard deviations and other measures reported;
- The values used to build graphs;
- The points extracted from images for analysis.

Authors do not need to submit their entire data set if only a portion of the data was used in the reported study.

If your submission does not contain these data, please either upload them as Supporting Information files or deposit them to a stable, public repository and provide us with the relevant URLs, DOIs, or accession numbers. For a list of recommended repositories, please see <https://journals.plos.org/plosone/s/recommended-repositories>.

If there are ethical or legal restrictions on sharing a de-identified data set, please explain them in detail (e.g., data contain potentially sensitive information, data are owned by a third-party organization, etc.) and who has imposed them (e.g., an ethics committee). Please also provide contact information for a data access committee, ethics committee, or other institutional body to which data requests may be sent. If data are owned by a third party, please indicate how others may request data access.

Response:  We attached our data as a supporting file. Please check.

**Additional Editor Comments:**

Kindly revise your manuscript extensively according to the attached reviewers' comments.

Response: We tried to revise the manuscript according to reviewers’ comments.

Add updated references and ensure that they follow the journal's guidelines.

Response: Tried to update the reference and style according to journal guidelines.

Extensive language and grammar revision is required.

Response: Tried to revise the language of the whole manuscript.

**Reviewers' comments:**

**Response to Reviewer's comments**

**Comments to the Author**

1. Is the manuscript technically sound, and do the data support the conclusions?

The manuscript must describe a technically sound piece of scientific research with data that supports the conclusions. Experiments must have been conducted rigorously, with appropriate controls, replication, and sample sizes. The conclusions must be drawn appropriately based on the data presented.

Reviewer #1: Partly

Reviewer #2: Partly

Reviewer #3: Yes

Reviewer #4: Yes

Reviewer #5: Yes

Reviewer #6: No

2. Has the statistical analysis been performed appropriately and rigorously?

Reviewer #1: I Don't Know

Reviewer #2: No

Reviewer #3: Yes

Reviewer #4: Yes

Reviewer #5: Yes

Reviewer #6: No

3. Have the authors made all data underlying the findings in their manuscript fully available?

The PLOS Data policy requires authors to make all data underlying the findings described in their manuscript fully available without restriction, with rare exception (please refer to the Data Availability Statement in the manuscript PDF file). The data should be provided as part of the manuscript or its supporting information, or deposited to a public repository. For example, in addition to summary statistics, the data points behind means, medians and variance measures should be available. If there are restrictions on publicly sharing data—e.g. participant privacy or use of data from a third party—those must be specified.

Reviewer #1: Yes

Reviewer #2: No

Reviewer #3: Yes

Reviewer #4: Yes

Reviewer #5: Yes

Reviewer #6: No

4. Is the manuscript presented in an intelligible fashion and written in standard English?

PLOS ONE does not copyedit accepted manuscripts, so the language in submitted articles must be clear, correct, and unambiguous. Any typographical or grammatical errors should be corrected at revision, so please note any specific errors here.

Reviewer #1: Yes

Reviewer #2: No

Reviewer #3: No

Reviewer #4: Yes

Reviewer #5: Yes

Reviewer #6: No

**Reviewers Comments to the Author**

**Reviewer #1:**

**Comment 6:** The manuscript entitled "Green synthesis and characterization of silver nanoparticles and its efficacy against Rhizoctonia solani, a fungus causing sheath blight disease in rice" has been reviewed.

The overall manuscript is good. However, before publishing it, there are several issues should be resolved. There are several minor grammatical errors, typo errors have been identified.

Response:  Thank you for your nice comments. The grammatical errors as well as typo errors existing in the preliminary manuscript was reviewed and solved throughout the corrected manuscript.

**Comment 7:** The abstract needs some modification.

Response: The abstract was modified accordingly. Page: 2

**Comment 8:** In the material and methods section, some of the methodology needs detailed description.

Response: In the material and methods section, methodology part was revised with detail explanation especially in 2.2 and 2.6 section. Page: 5 & Page 7.

**Reviewer #2:**

We are very much grateful for your valuable comments. We tried to answer your queries in the following section

**Comment 9:** The English language of the manuscript is very poor. It must be improved.

Response: We tried to revise the language of our manuscript. The manuscript has been edited by Dr. Mohammad Abdul Latif, Director, Admin and Common Service and Former Head and Chief Scientific Officer of the Plant Pathology Division of Bangladesh Rice Research Institute (BRRI), Gazipur, Bangladesh.

**Comment 10:** The abstract is too long; it should be shortened and the abbreviations should not be mentioned in the abstract. For example, the genera of organisms should be written in full.

Response: The abstract was modified accordingly. Page: 2.

**Comment 11:** AgNO3 is correct to AgNO_3_ throughout the manuscript.

Response: An accurate form of AgNO3 was placed throughout the manuscript.

**Comment 12:** The paragraph 2.2. is confusing and must be modified for understanding.

Response: The paragraph 2.2 was rewritten more elaboratively for clear understanding. Page: 5.

**Comment 13:** The authors did not explain the characteristics of the soil used in the pot experiment

Response: In the pot experiment we used sterilized alluvial loamy soil which was not reported as toxic to any crop before. However, we have a control treatment as well through which we can extract the key effect of sole Ag nanoparticles in the experiment. Page: 7.

**Comment 14:** The paragraph 2.6 is confusing and must be modified for understanding.

Response: The paragraph 2.6 was rewritten accordingly. Page: 7.

**Comment 14:** Line 303: What are the authors mean by the 3-day old mycelial blocks *R. solani* were placed in the center of the plants?

Response: We used 3-day-old mycelial blocks of *R. solani* to inoculate the rice plants. It means young culture (3 days old) of sheath blight pathogen was used for pathogenicity test. Youn culture of *R. solani* are more active to cause disease initiation so that the efficacy of the used nanoparticles would be more distinguishable.

**Comment 15:** Dholkolimi or dholkolimi, please unify throughout the manuscript.

Response: Dholkolmi is unified throughout the manuscript. Page: 2, 5, 6, 7, 8, 9, 10, 11 etc.

**Comment 16:** Amistar or amister?

Response: Author replaced amister with Amistar (the correct spelling and wording) throughout the manuscript.

**Comment 17:** In vivo or in vitro should be written in italic.

Response: Author replace both in vivo or in vitro in italic form throughout the manuscript.

**Comment 18:** The color or colour, please unify throughout the manuscript.

Response: Author used color instead of colour through- out the manuscript.

**Comment 19:** Min. or minutes, please unify throughout the manuscript.

Response: Author used minutes in throughout the manuscript. Page: 5, Page: 8.

**Comment 20:** The author must follow the style of the journal during writing particularly the references list.

Response: The author corrected the bibliography style according to the journal style. Page 17-25.

**Reviewer #3**:

**Comment 21:** Figure 2 a make spectra in a cristanality form like 100 ,101, 201 etc and have compared the data with JCPD number.

Response: Thank you for your valuable comments. The author corrected the Figure 2a accordingly. Please check Figure 2a.

**Comment 22:** FTIR graph overlaying with standard silver nitrate graph and check it starts from higher range to lower 4000 to 600.

Response: Author modified the FTIR graph with standard silver nanoparticles along with leaf extracts. Here, the range exists between 4000-450 cm^-1^. (Figure 2 b).

**Comment 23:** XRD shows it is not in a pure form it contains C, O, Cl also and explain about atoms percentage in graph and Ag is less.

Response: EDX peaks revealed C, O, Al and may be due to the carbon-coated copper grid with aluminum stub, and Cl might be come from leaf extract.

The weight percentage of an element is the weight of that element measured in the sample divided by the weight of all elements in the sample multiplied by 100.

The atomic percentage is the number of atoms of that element, at that weight percentage, divided by the total number of atoms in the sample multiplied by 100.

So, the atomic weight percent is calculated from the element weight percentage by dividing each element weight percentage by its atomic weight, do this for all elements in the sample, you will have a list of atomic proportions. Sum these together to obtain a total atomic weight. Then for each element in the sample divide its atomic proportion by the total and * 100.

Here is an example -. EDX will give you

C: el wt% 14 O: el wt% 10.9 Cl: el wt% 5.84 Ag: el wt% 68.38

divide each by their atomic weights C (12), O (16), Cl (35.5), Ag (107.868) and sum

C: 14 / 12 = 1.16 O: 10.9/16 = 0.68 Cl: 5.84/35.5 = 0.16 Ag: 68.38/ 107.868= 0.63

divide each by the sum (2.63) and turn into atomic percentage

Ag: 0.63/ 2.63 * 100 = 24.1(23.33±1.06) at atomic wt%

**Comment 24:** for all UV graphs there is a peak in UV range what it indicates, and why u have taken control as pH 5.5

Response: UV- vis spectrometer was used for the primary characterization of nanoparticles. This peak indicates the presence of AgNPs. In this study, the effect of pH was studied in two different conditions including fresh leaf extract (pH-5.8) and alkaline conditions.

**Comment 25:** References are not in same style.

Response: The author corrected the bibliography style according to the journal style. Page 17-25.

**Comment 26:** have you taken any standard pesticide as a control for comparison?

Response: The author took a standard pesticide (Amistar top) as a control for comparison.

**Reviewer #4:**

A good manuscript with sound information. However, some improvement is needed before it gets published.

Response: We are very much grateful for your valuable comments. We tried to answer your queries in the following section

**Comment 27:** Please signify the novelty of the work. Developing AgNPs via plant sources and observing their anti-microbial activity is not new. Please indicate the novelty of your work given plant used, process used, or fungi used in the exp.

Response: In this study, antifungal efficacy was evaluated against *R. solani* using Dholkolmi mediated AgNPs. AgNPs were also evaluated under field conditions as liquid form. Dholkolmi mediated synthesis of Ag nanoparticles and their characterization using different method and testing the efficacy of synthesized AgNPs against rice sheath blight pathogen are the novelty of our works.

**Comment 28:** Please mention the solution used to increase the pH of plant extract for developing AgNPs.

Response: The author used KOH to increase the pH of plant extract for developing AgNPs. Page: 5; Line- 153.

**Comment 29:** Why pH 7 and 8 are missing? Do you have any specific reason behind this?

Response: The effect of pH was studied in two different conditions including fresh leaf extract (pH-5.8) and alkaline conditions (pH-9, 10 & 11). We have no specific reason to avoid pH 7 and 8 because we know that higher pH influences the chemical reaction compared to a neutral pH range like 6 to 8,so we directly used pH 9, 10 and 11.

**Comment 30:** FTIR data of plant extract is missing, if possible, please add the same.

Response: The author included FTIR data of plant extract along with AgNPs in Figure-2b.

**Comment 30:** Please improve the discussion part.

Response: We tried to improve the discussion of our manuscript. The discussion has been edited by Dr. Mohammad Abdul Latif, Director, Admin and Common Service and Former Head and Chief Scientific Officer of the Plant Pathology Division of Bangladesh Rice Research Institute (BRRI), Gazipur, Bangladesh.

**Reviewer #5:**

The manuscript entitled:" Green synthesis and characterization of silver nanoparticles and its efficacy against Rhizoctonia solani, a fungus causing sheath blight disease in rice" has been reviewed. In my opinion, it’s very interesting and falls really well with the scope of the journal. So, I recommend it to be accepted.

Response: Thank you for your nice comments.

**Reviewer #6:**

**Comment 31:** The manuscript is technically week and needs major improvements.

Response: We are very much grateful for your valuable comments. We tried to improve the manuscript.

**Comment 32:** The figures are very poorly taken, not appropriate, and unable to read. there are many statistical errors and typo mistakes. Whole manuscripts need to be rewritten.

Response: May be resolution becomes low due to upload in the journal system or in the pdf file which is sent to you. However, we tried to improved figures. We also corrected the typo mistakes and tried to improve the language of the manuscript.
